# Quality Control Test for Sequence-Phenotype Assignments

**DOI:** 10.1371/journal.pone.0118288

**Published:** 2015-02-20

**Authors:** Maria Teresa Lara Ortiz, Pablo Benjamín Leon Rosario, Pablo Luna-Nevarez, Alba Savin Gamez, Ana Martínez-del Campo, Gabriel Del Rio

**Affiliations:** 1 Department of Biochemistry and Structural Biology. Instituto de Fisiología Celular at the Universidad Nacional Autónoma de México, México DF, 04510, México; 2 Department of agronomical sciences and veterinary. Sonora Institute of Technology, Obregon city 85000, Mexico; 3 Department of Genetics. Instituto de Fisiología Celular at the Universidad Nacional Autónoma de México, México DF, 04510, México; NIDCR/NIH, UNITED STATES

## Abstract

Relating a gene mutation to a phenotype is a common task in different disciplines such as protein biochemistry. In this endeavour, it is common to find false relationships arising from mutations introduced by cells that may be depurated using a phenotypic assay; yet, such phenotypic assays may introduce additional false relationships arising from experimental errors. Here we introduce the use of high-throughput DNA sequencers and statistical analysis aimed to identify incorrect DNA sequence-phenotype assignments and observed that 10–20% of these false assignments are expected in large screenings aimed to identify critical residues for protein function. We further show that this level of incorrect DNA sequence-phenotype assignments may significantly alter our understanding about the structure-function relationship of proteins. We have made available an implementation of our method at http://bis.ifc.unam.mx/en/software/chispas.

## Introduction

The study of protein structure-function relationship involves the identification of residues indispensable for protein function (critical residues); critical residues are commonly identified as those positions in proteins that result in loss of protein activity by affecting the proper protein folding, protein stability and/or the ability to perform a biochemical activity [1]. As a consequence, many protein coding genes have been subjected to site-directed mutagenesis experiments in the past with the aim of identifying the protein critical residues [2,3] and such information has been used to develop prediction methods useful to test our understanding about the function of these residues in proteins [4,5]. Alternatively, directed evolution experiments circumvent our limitations to understand the structure-function relationship of proteins by discovering protein variants with valuable features [6]. In either case, it is important to validate the identity of the mutated residues to guarantee the reproducibility of the results and to reduce any bias on methods aimed to predict critical residues. The nature of mutations affecting protein function is established by sequencing the corresponding protein-coding DNA region. The relevance of the presence of DNA variants in a population for critical residue identification became apparent when noticing that combining two or more protein variants may render a mutant phenotype [7]. Furthermore, in the presence of a selective condition that may have been used to screen for protein variants (*e*.*g*., an antibiotic that would kill any cell expressing a protein with wild-type activity), it is expected that natural variants could mask the true relevance of a given residue for protein function. One way to test for the presence of false critical residue assignments is to search for protein variants presenting both wild-type and mutant phenotypes. For instance, in the mutagenesis of T4 lysozyme [8] and beta-lactamase [9] several residues identified as critical (found in cells expressing protein variants with an altered phenotype) were also found in cells with a wild-type phenotype, indicating that these were false critical residues; noticeable, 33 out of 78 identified critical residues in the dihydrofolate reductase were false critical residues [10]. Besides saturation mutagenesis experiments, altering the codon usage (e.g., by site-directed mutagenesis) in a protein coding gene might as well render cells expressing more than a single protein with different activities [11]. Yet, identifying the same gene mutation in cells presenting more than one phenotype may as well be explained by errors introduced by the methodology used to screen for them. Thus, while a simple phenotypic screening may help testing for the validity of some critical residue assignments, other false positive critical residues derived from methodological errors require further analysis.

Here, we introduce an experimental method aimed to identify the DNA variants incorrectly assigned to a phenotype derived from both experimental errors and natural variations; we will refer to these as incorrect sequence-phenotype assignments or simply ISPAs. Hence, our method aimed to CHeck for ISPAs is referred to as CHISPAs. We applied CHISPAs to validate the identification of critical residues of HokC, a transmembrane protein from *Escherichia coli*, and showed that 12% or up to 20% of the identified critical residues were ISPAs, depending on the statistical model used. A reliability test on predictors of critical residues provided an independent way to assess the impact to include this proportion of ISPAs in our understanding of the structure-function relationship of HokC.

## Material and Methods

### Strains and Reagents

The bacterial strains used in our studies were *Escherichia coli* MC4100 Δ(argF-lac)U169 araD139 rpsL150 relA1 flbB5301 deoC1 ptsF25 rbsR; *E*. *coli* XL1-Blue supE44 hsdR17 recA1 endA1 gyrA96 thi-1 relA1 lac-; *E*. *coli* DH5α supE44 ΔlacU169 (φ80 lacZ ΔM15) hsdR17 recA1 endA1 gyrA96 thi-1 relA1.

The plasmid pEXT22/frg-hokC containing the gene *hokC* starting at the second ATG was used as template for both PCR random mutagenesis and for the site-directed mutagenesis.

### Mutagenesis

Two strategies were performed in this study. The first one, random mutagenic PCR [12], was used to test for the presence of natural variations during the selection of cells surviving to *hokC* over-expression. Briefly, we used two oligo-nucleotides designed to amplify the coding region of *hokC*:

1. Oligo EcoRI 5´ AAC AAT TTC ACA CAG GAA ACA GAA TTC 3’
2. Oligo HindIII 5´ CGC CCG CCA TAA ACT GCC AAG C 3’

Where Oligo EcoRI and Oligo HindIII were used to introduce EcoRI and HindII restriction sites, respectively.

To induce mutations during the amplification of *hokC*, we used a bias composition of deoxy-nucleotides (0.2 mM dATP, 0.2 mM dGTP, 1 mM dTTP and 1 mM dCTP), 3 mM MgCl2, 0.3 μM each primer and 5 units of Taq Polymerase in a 50 μl total volume.

Alternatively, site-directed mutagenesis on the coding region of HokC trans-membrane region was performed using the QuikChange Site-Directed Mutagenesis Kit (Agilent Stratagene, USA). The following libraries of oligonucleotides were used for this goal:

Oligo R5 F 5´ CCT GAT CGT CAT CTG T SNS SNS SNS GTA GTG GCG G 3’

Oligo R5 R 5´ CCG CCA CTA C SNS SNS SNS ACA GAT GAC GAT CAG G 3’

Where S stand for G or C nucleotides and N for any of the four nucleotides. These oligonucleotides mutate 3 residues (Ile16, Thr17 and Ala18) in the middle part of the trans-membrane coding region of *hokC*. Note that these oligonucleotides will generate mutant codons with SNS composition coding for 10 (L, P, H, Q, R, V, A, D, E, G) out of the 20 conventional amino acid residues. In this way, the number of variants to be screened is reduced and at the same time keeping the diversity of physicochemical properties of the amino acid residues.

For the site-directed mutagenesis reactions we followed the instructions of the manufacturer: 50 ng of plasmid (pEXT22/frg-hokC), a pair of mutagenic oligonucleotides (125 ng), 1 μl dNTP mix, 5 μl of 10x reaction buffer and 2.5 U of Pfu Turbo DNA Polymerase in a 50 μl total volume.

### Sub cloning and Transformation

5 clones obtained from each mutagenic PCR were selected to test the presence of spontaneous mutations. The plasmids were purified using the QIAprep MiniPrep kit (QIAGEN, USA) and digested with EcoRI and HindIII restriction enzymes (Invitrogen, USA) following the provider specifications.

*E*. *coli* cells were transformed with plasmids harbouring the gene of interest using a method previously described [13].

### Selection of clones

To sequence the *hokC* variants with wild-type and mutant phenotypes, we performed the following procedure. *E*. *coli* cells were grown in Luria broth with kanamycin to select for those carrying the plasmid expressing *hokC* mutations. The plasmid, pEXT22, includes a non-leaky promoter induced by IPTG [14]. The over-expression of *hokC* was achieved by adding IPTG to the media; this would kill cells expressing a wild-type-like HokC activity. However, cells expressing a mutation critical for HokC activity will grow. The chromosomal copy of *hokC* has 3 ATG codons; we noticed that over-expression of the ORF including the 3 ATG codons did not kill all cells; on the contrary, the *hokC* gene expressed from the second ATG found in that ORF had more toxic effect on *E*. *coli* cells (data not shown). Therefore, all our mutagenesis experiments were performed on this short version of *hokC*. To select colonies for sequencing, we looked for isolated colonies; for that end, we used large plates (245 mm x 245 mm of area).

### Sequencing

To evaluate the presence of ISPAs, we used the services of the genomic services unit from CINVESTAV-LANGEBIO, México. Briefly, we generated two 96-well plates filled with the bacteria culture of interest in 10% glycerol; one plate contained the clones with a mutant phenotype isolated in the presence of IPTG and the other in the absence of it. From these samples, the plasmidic DNA carrying *hokC* was extracted and sequenced using the Sanger method in the capillary systems provided by ABI 3730 (Applied Biosystems) and MegaBACE 4500 (GE Healthcare) rendering about 400 nucleotides in each read; since *hokC* is 150 nucleotide long, this sequencing allowed to determine the presence of mutations inside and outside the open reading frame of *hokC*.

To sequence mutants in the middle of the trans-membrane coding region of *hokC*, we implemented the following procedure. Colonies with wild-type or mutant phenotypes were picked and grown overnight in 3 ml of LB media with kanamycin. These colonies were pooled in 2 groups according to their origin: cells with a wild-type and mutant phenotypes. From these pools, DNA was extracted. Thus, two pools of plasmids were obtained: from wild-type and mutant phenotype colonies. Note that this pooling is required since sequencing thousands of colonies would not be practical. From these DNA molecules, the mutated *hokC* region was amplified by PCR; the final size of the PCR product was 376 bp. This sample was mixed at equimolar ratios and sequenced at the “Unidad Universitaria de Secuenciación Masiva de DNA-UNAM” using the Genome Analyzer System GAIIx from Illumina company. Since this sequencer has the capacity to generate 10^7^ DNA reads and the number of bacterial colonies to be sequenced is substantially smaller than this number (10^3^), the experiment could generate thousands of clusters with exactly the same sequence. In such case, the equipment may not be able to identify this experiment as valid. To prevent this from happening, the sequencing mixtures were contaminated with genomic DNA from *E*. *coli*. While *hokC* is on the genome of *E*. *coli*, note that genomic DNA may be differentiated from the mutations generated in our procedure by the composition of the mutated codons (SNS, see section Site Directed Mutagenesis above) and the 5’ and 3’ ends.

### Statistical analysis

Our work describes an experimental procedure aimed to identify incorrect sequence-phenotype assignments (ISPAs) using high-throughput DNA sequencers. While ISPAs may occur in single point site-directed mutagenesis experiments, their identification is more relevant in large-scale mutagenesis experiments. In such large screenings, ISPAs are identified as those identical DNA sequences isolated from cells presenting different phenotypes (mutant and wild-type). Yet, it is common practice in these large experiments to sequence only the protein-coding region of the gene of interest, thus ignoring possible mutations that may alter the expression/function of the gene or protein of interest that are commonly found outside of the protein-coding region (*e*.*g*., promoter region); such mutations may mask the true effect of the mutation in the protein coding region and consequently affect the identification of ISPAs. Thus, it is important to recognize that mutations may be introduced by cells and/or by errors introduced by the phenotype assignment protocol used and/or the DNA amplification/sequencing. While it is not practical to sequence whole genomes to identify mutations outside the protein-coding region in large-scale mutagenesis experiments, it is possible to estimate the frequency of ISPAs expected from the phenotype assignment protocol and/or the DNA amplification/sequencing. Thus, here we describe a method aimed to identify those ISPAs that may be explained by these experimental errors and at the same time to identify the ISPAs derived as a consequence of mutations introduced by cells.

**Fig 1:**
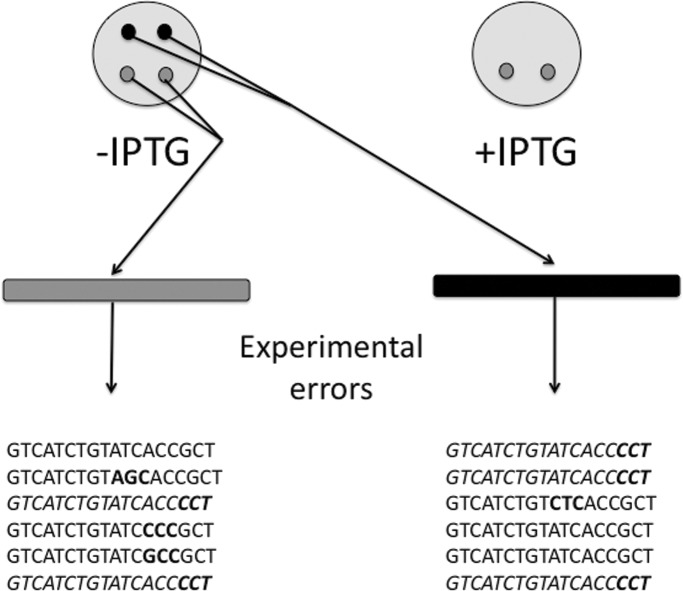
Sequencing procedure to identify ISPAs. The figure represents bacterial colonies in plates (big grey circles) that are induced to express (+) or not (-) *hokC*. Colonies presenting a wild-type and mutant phenotypes are depicted in black and light grey circles, respectively; bars represent the corresponding DNA molecules amplified from these bacterial colonies: black bar represent DNA molecules from cells presenting a wild-type phenotype. During this experimental procedure, it is expected that sequencing errors will be added to the errors introduced by the phenotype assignment; such errors may be reflected in incorrect relationships between DNA sequences and phenotypes. According to our method, if these experimental errors would be accumulated in DNA sequences from a given phenotype at a rate above the experimental errors these should be classified as ISPAs. The DNA sequences obtained by the sequencing procedure are represented at the bottom of the image, indicating in **bold** the mutated bases and in *italics* the sequences found in both phenotypes.

Provided that we have obtained DNA sequences assigned to wild-type and/or mutant phenotypes (see Fig. 1), then we need to identify the ISPAs. An ISPA is any DNA sequence that is found in both wild-type and mutant phenotypes. Here we recognize that ISPAs may be produced by mutations introduced by cells, experimental errors introduced by the phenotype assignment and/or the DNA amplification/sequencing, and propose a method to filter out ISPAs produced by experimental errors. Note that by filtering out ISPAs derived from experimental errors, our method may reduce the number of ISPAs in mutagenesis experiments and consequently this correction on ISPAs occurrence might have an impact on structure-function relationships studies (see below).

In our experimental setup, the number of DNA reads (*N*) should be related to the number of DNA sequences observed with only one phenotype (wild-type or mutant, here referred to as set *U*) plus the number of sequences observed with both phenotypes (wild-type and mutant, here referred to as set *B*). Note that any given sequence in set *U* or set *B* may be found multiple times in a high-throughput sequencing experiment (see formula 1), a condition necessary to identify ISPAs with statistical significance. For instance, lets say that in our sequencing procedure we identified only two different sequences: sequence 1 (*e*.*g*., CCC) may be found 50 times in both phenotypes, while sequence 2 (*e*.*g*., CTC) is found 150 times only in cells with wild-type phenotype; in such case, sequence 1 would be part of the *B* set and sequence 2 to the *U* set with their corresponding frequencies, *n* = 50 and *m* = 150 respectively (see formula 1).

$$N\geq \sum_{i=1}^{i=m}U_{i}+\sum_{j=1}^{j=n}B_{j}$$ (1)

Equality in (1) is satisfied only for non-identical sequences found in one or both phenotypes. ISPAs occurrence depends on the experimental errors (*E*, corresponding to errors in the sequencing procedure and/or the phenotype assignment procedure) and we expect these errors to be present in DNA sequences found in both *U* and *B* (see formula 2 and 3). To represent this idea, we used *e*_*U*_ and *e*_*B*_ as the fraction of sequences that include a mutation or incorrect phenotype assignment as a consequence of experimental errors in the *U* or *B* sets, respectively. Thus:

$$E+G=f(e_{U},e_{B})$$ (2)

$$N^{*}\left(E+G\right)\geq \sum_{i=1}^{i=m}U_{i}*e_{u}+\sum_{j=1}^{j=n}B_{j}*e_{B}$$ (3)

Note that in formulas 2 and 3, *G* corresponds to the variation from the average *E* values obtained from experimental observations (see below on how to determine this value).

The relation in (2) establishes the criterion to identify ISPAs derived from experimental errors and claims that *E* + *G* is a function of the errors derived from assigning any sequence to a phenotype and/or the amplification/sequencing of DNA. Hence, a quality assessment of the sequencing procedure can be established when the following inequity is satisfied:

$$N^{*}\left(E+G\right)\geq \sum_{j=1}^{j=n}B_{j}*e_{B}$$ (4)

Note that *N*, *E* and *e*_B_ can be obtained from experimental data. Since it is not possible to estimate *e*_*U*_ from DNA sequencing data alone, it is necessary to estimate it independently of DNA sequencing data as described in formula 4; hence the relevance to estimate *E* and its dispersion (*G*) is to establish the size of set *B* that is expected from experimental errors. We describe two different approaches to estimate *G*:

1. Binomial model (**BiM**): estimate *G* assuming that this corresponds to the upper limit of the confidence interval at a given α error according to Agresti and Coull approximation to a binomial distribution [15]. In such case:

$$G=p^{\prime }\pm z_{1+\frac{1}{2}\alpha }\sqrt{\frac{1}{n^{\prime }}p^{\prime }\left(1-p^{\prime }\right)}$$ (5)

Where *n*’ is the corrected number of trials (*n*’ = *n*+ z^2^_1–1/2α_), *p*’ is the corrected number of successes (p’ = (1/n’)*(p+0.5* z^2^_1–1/2α_)) and z_1–1/2α_ is the z value of a binomial distribution for a given α error value. We chose the Agresti and Coull approximation because the expected *E* value is small. In our case, the *G* value associated to the leakiness (see Methods) of the selection method (*G*_Leakiness_) can be estimated experimentally; the *G* value associated to the sequencing procedure (*G*_Sequencing_) if unknown may be assumed to be equals to 0. In such case, *G* = *G*_Leakiness_.

1. Bayes model (**ByM**): it combines two sources of information, a prior distribution π(Ф) that describes our beliefs about the estimated parameter for *e*_B_, and a density model f(x|Ф) that describes the distribution of data x given Ф, which is called the likelihood function. As result, a posterior distribution π(Ф|x) is calculated to estimate the expected distribution of *e*_B_ using Bayes theorem as follows:

$$\pi \left(\phi |x\right)=\frac{f\left(x|\phi \right)\pi \left(\phi \right)}{\pi \left(x\right)}$$ 6

Interval estimation from Bayesian analyses calculates the credible intervals. The highest posterior density (HPD) interval is a frequently used Bayesian credible set, and the 100(1-α)% means that there is a 95% chance that the parameter *e*_B_ is in this interval when α = 0.05.

Thus, in the first model (BiM) we assume a binomial distribution considering that the phenotype assignments are binary and the error is modelled using the Agresti and Coull approximation. In the second case we assume a binomial model where the phenotype assignment is based on a probability distribution. While any of these assumptions are reasonable, we explored these two models and performed an independent reliability test to evaluate the implications of any of these models in our understanding of the structure-function relationship of HokC.

To explain our method, lets assume that after a mutagenesis experiment we identified a group of sequences that are found in cells with both a wild-type and a mutant phenotype; for this example a given sequence may be found 30 times in a wild-type phenotype and 300 in a mutant phenotype; such sequence corresponds to an ISPA. This ISPA is found 10% of the cases in the wild-type phenotype; if the experimental error is found to be less than 10%, then the ISPA in this example may be considered a true ISPA (ISPA derived from mutations introduced by cells), otherwise it should be considered a false ISPA (ISPA derived from experimental errors). Note that in order for this method to work, it is necessary to have multiple instances of the same DNA sequence found in both phenotypes; to generate this data we propose to use high-throughput DNA sequencers.

Thus, to identify the DNA sequences that have been incorrectly related to a phenotype, our method requires data from two experimental sources (see Fig. 1):

1. **Phenotype assignment**. This implies to identify cells presenting a wild-type or a mutant phenotype.
2. **DNA high-throughput sequencing**. This requires extracting DNA from these cells and differentially tagging them [18] to perform high-throughput sequencing.

From these procedures a collection of DNA sequences can be obtained from cells presenting a wild type and/or a mutant phenotype. In the Methods section, a statistical procedure aimed to treat the experimental results is described.

### Data analysis of DNA reads

The sequenced products were stored in a structured database (MySQL) to identify the ISPAs using a program written in Java language developed for this purpose that it is available from our website http://bis.ifc.unam.mx/en/software/chispas; this code verifies that the sequence includes the wild-type flanking regions of *hokC* and in between has the 3 codons with SNS composition. Then, the sequences of the mutated regions were grouped and their frequencies in both wild-type and mutant phenotypes were determined automatically by our code.

In every selection procedure some false positives may be expected; we referred to these false positives as the leakiness of the selection procedure. For the statistical analysis to determine the confidence interval of the leakiness of the selection method, we used the R implementation of the Agresti and Coull method, binom.confint. For the Bayes analysis we used the SAS implementation through the GENMOD procedure for logistic regression (Version 9.2; SAS Inst. Inc., Cary, NC) and the scripts are available from the authors upon request.

In the BiM, the expected number of ISPAs per sequence is derived by adjusting the observed binomial media (μ = 0.062) and standard deviation (σ = [p*q/N]0.5) from experimental errors to a normal distribution; this normal distribution is compared against the Z-score (Z = (x-μ)/σ) of the less frequently observed phenotype for any given sequence. In our case, for an error α = 0.05, any Z< = 1.65 includes incorrect phenotype assignments for a given sequence that may be explained by the expected experimental/technical errors.

### Reliability analysis of critical residues predictors

**Fig 2:**
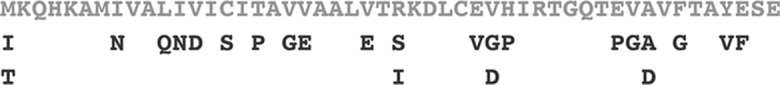
Single-point mutations of HokC. The figure shows in grey colour the amino acid wild-type sequence of HokC; below the single point mutations identified with a mutant phenotype (surviving to the over-expression of *hokC*).

To estimate the impact on the reliability of prediction methods by changing the list of critical residues, we used HokC and the HIV-1 protease. In the case of HokC, the 20 residues identified in this report to be critical for HokC activity (see Fig. 2; positions 1, 8, 11,12, 13, 15, 17, 19, 20, 24, 26, 31, 32, 33, 40, 41, 42, 44, 47, 48) were added to those critical residues previously reported (see Table A in S3 Data) giving a total of 25 critical residues, including those at positions 1, 2, 8, 11, 12, 13, 15, 17, 19, 20, 24, 25, 26, 28, 29, 31, 32, 33, 39, 40, 41, 43, 46, 47 and 49. In the case of the HIV-1 protease, 46 residues have been reported to be critical for the function of this protease [16], including residues at the following positions: 2, 5, 9, 13, 15, 22–29, 31–33, 36, 40, 46, 47, 49–52, 56, 57, 59, 62, 65, 68, 74–81, 83–90 and 97; these residues were considered positives (P) and the rest were considered negatives (F); note that mutations at a conserved position only included 23 residues at positions 9, 15, 23–29, 31–33, 47, 49, 51, 52, 74, 81, 84, 86, 87, 90 and 97. Then, 20% of these residues were randomly assigned as negative predictions and this was done 100 times. Four predictors were used for this study: ConSurf, a sequence-based predictor [17]; POOL, a structure-based predictor [18]; a random and a perfect predictors. For this last one, we simply used the same list of known critical residues as the predicted critical ones; for the random predictor, we randomized the list of residues using a Java code available from the authors upon request. Each one of these predictors generated an ordered list of residues; from this list, we picked the top 5, 10, 15 and up to 95% of all the residues and calculated the true positives (TP), true negatives (TN), false positives (FP) and false negatives (FN). Then, we obtained three statistical parameters to determine the reliability of these predictors for the 101 groups of top predicted residues: sensitivity (TP/P), specificity (TN/F) and Matthews correlation coefficient or MCC ([TP*TN—FP*FN]/[(TP+FP)*(TP+FN)*(TN+FP)*(TN+FN)]). All this procedures were done by Java codes developed for this work and are available from the authors upon request.

## Results

### Identification of critical residues on HokC

We performed mutagenesis experiments on *hokC*, an *Escherichia coli* gene that codes for a protein that induces the killing of its host upon expression [19]; in this case, cells expressing a wild-type copy of *hokC* should die upon its expression (wild-type phenotype) and those expressing mutated copies of *hokC* at a critical residue should survive (mutant phenotype). From a random PCR-based mutagenesis several new critical residues for HokC (see Fig. 2) and spurious mutations (wild-type *hokC* sequences rendering a mutant phenotype presenting mutations at the promoter region; see Table 1) were identified. As expected, mutations outside the *hokC* open reading frame were more frequently found in the presence of the selection condition (12 out 94 colonies sequenced or 12.7%) than in its absence (1 out of 94 colonies sequenced or 1%). In the absence of a selection condition, few if any mutations introduced by cells are expected, hence most of mutations outside the *hokC* open reading frame may be derived from experimental errors in the sequencing and/or the phenotype assignment protocols and some other may as well be found in the open-reading frame of *hokC*. To test this idea, we applied CHISPAs to an independent mutagenesis of *hokC*.

**Table 1:** Spurious mutations in the promoter region of *hokC*.

	-35	-10
Tac promoter	TTGACA	TATAAT
Mut A05	TgGcCA	TAaAAT
Mut B10	TcGACA	TATAAT
Mut C05	T-GACA	TATAAT
Mut M18	TTGggA	—————-
Mut F01	TTGACA	TATAAc
Mut B05	TTGACA	TATAAc
Mut C02	TTGACA	TtTccT
Mut B09	TTGACA	TATAAc
Mut B07	—————-	—————-
Mut A03	—————-	—————-
Mut E02	—————-	—————-
Mut B01	—————-	TATAAT
Mut C04	—————-	cATgA-
Wt C08	cTcaAgA	TgcAtcG

Mutations on the promoter region are shown in lower case letters and deletion with dash symbol. DNA sequences isolated from colonies presenting mutant phenotype (Mut XXX) or wild-type phenotype (Wt C08) are shown.

### Identifying Incorrect Sequence-Phenotype Assignments

Site-directed saturation mutagenesis on a three-residue region in the transmembrane region of HokC (Ile16, Thr17 and Ala18) was performed; this region includes a conserved residue, Thr17, and is located in a region previously assumed not to play a critical role in the function of HokC [20]. From this mutagenesis experiment, we sequenced the DNA isolated from 945 *E*. *coli* colonies expressing *hokC* with wild-type (246 colonies) or mutant phenotypes (699 colonies). Considering the expected enrichment of mutations (*e*.*g*., adaptive mutations) in the presence of a selection condition, only the bacterial colonies grown in the absence of the selection condition were sequenced; 353197 sequences were obtained from high-throughput sequencing (the raw data may be obtained from the authors upon request). These correspond to 844 unique sequences with wild-type phenotype and 842 unique sequences with mutant phenotype (see Tables B and C in S3 Data). Note that this relatively small number of sequences obtained from the high-throughput sequencer was required to allow for the equipment to correctly identify the clusters of DNA molecules (see Methods).

**Fig 3:**
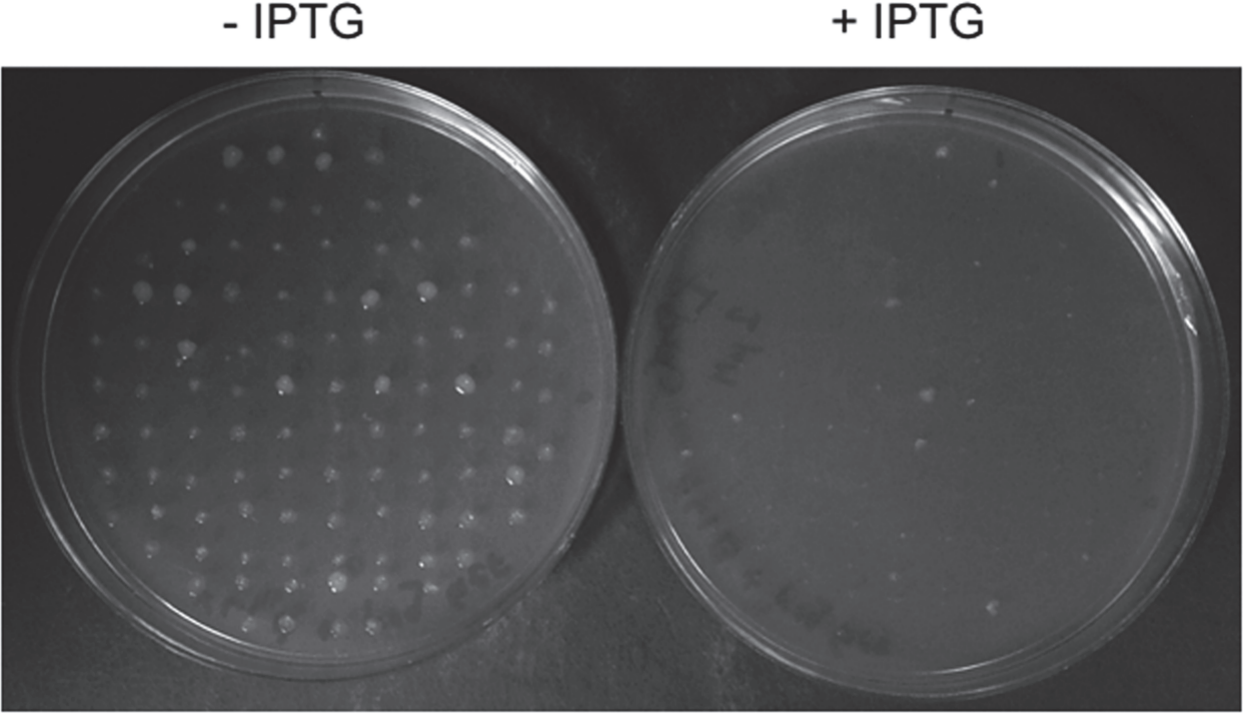
Quantifying the leakiness of the selection method. The photo shows a plate where 100 colonies of *Escherichia coli* cells transformed with a plasmid harboring the wild type *hokC* gene (pEXT22-frg-*hokC*; see Methods) were exposed to IPTG to induce *hokC* expression. The wild type phenotype corresponds with the absence of cell growth in the presence of IPTG, while a mutant phenotype corresponds with cell growth in the presence of IPTG. In a non-leaky system no mutant phenotype should be observed, yet this image shows the presence of 6 colonies growing in the presence of IPTG. This experiment was repeated 4 times.

We assumed G_Sequencing_ = 0 considering that the error rate associated with the sequencing process by Illumina high-throughput sequencers is estimated to be less that 1% [21]. We determined the rate of false positives observable in our selection method, herein referred to as leakiness, by analysing 4x100 *E*. *coli* colonies expressing a wild-type *hokC* gene and exposed them to a selection condition (see Methods); we found that in 3% of the cases (1.6%-5.2% at 95% confidence interval) a wild-type copy of *hokC* expressed in *E*. *coli* rendered a mutant phenotype (see Fig. 3). As expected, this rate of errors in phenotype assignments in the absence of a selection condition is smaller than the one observed in the presence of a lethal condition (see above). According to the BiM, the upper limit in assigning 3% as the error by the leakiness of the screening method is 5.2% at 95% of confidence, thus *G*_Leakiness_ = 2.2%. In the case of ByM, *G*_Leakiness_ is derived from the average of the HPD interval; here, the upper limit was considered only for positive *e*_B_ values according to the posterior distribution. We obtained 4.0% as the upper limit in assigning 3% of errors in the phenotype assignments with the Bayesian model with 95% of confidence, hence *G*_Leakiness_ = 1.0%.

Overall, the expected rate of DNA variations by experimental/technical reasons (*E+G*) in our models (BiM or ByM) varies and hence there would be differences in the ISPAs identified by any of these procedures. Accordingly, using the BiM, 328 different sequences annotated with both phenotypes can be explained by experimental/technical errors, which represents 27.06% of the 1212 different sequences obtained from the sequencing experiment (see Tables B and C in S3 Data) or 69.05% of the 475 unique sequences found with both wild-type and mutant phenotypes (see Table D in S3 Data); thus 69% of the ISPAs are indeed false ISPAs because they can be explained by experimental errors. By contrast, using the ByM, 248 out of 475 (52%) unique sequences found with both wild-type and mutant phenotypes (see Table D in S3 Data) have a sequence-phenotype assignment that can be explained from the experimental/technical errors. Therefore, BiM identified 147 true ISPAs (12%) while ByM identified 248 true ISPAs (20%). From these results, we obtained the common ISPAs identified by both models and found 145 unique sequences (see Table D in S3 Data). These 145 ISPAs correspond to 11.96% of all unique sequences assigned to a phenotype.

### Effect on critical residue prediction by ISPAs

**Fig 4:**
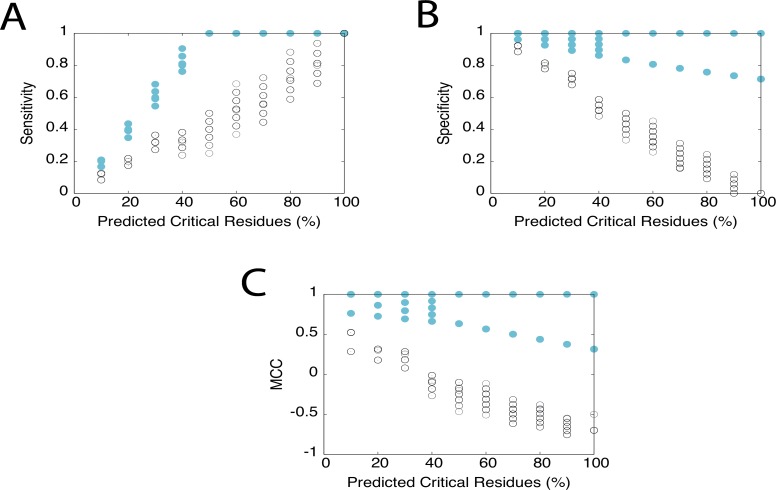
Effect on the accuracy of a perfect prediction of critical residues in HokC by considering ISPAs. Prediction of the known critical residues of HokC is shown for a perfect predictor (filled cyan circles) and a random predictor (open black circles). The different circles correspond to the variation on the reliability of these predictions when 20% of the 25 critical residues identified in this and previous studies were considered ISPAs (see Methods). These predictors generate an ordered list of residues and the x-axis indicates the percentage of critical residues taken from the top of these lists. A) Plots in the Y-axis the sensitivity, B) plots in the Y-axis the specificity and C) plots in the Y-axis the Mathews Correlation Coefficient. The image was generated using gnuplot.

**Fig 5:**
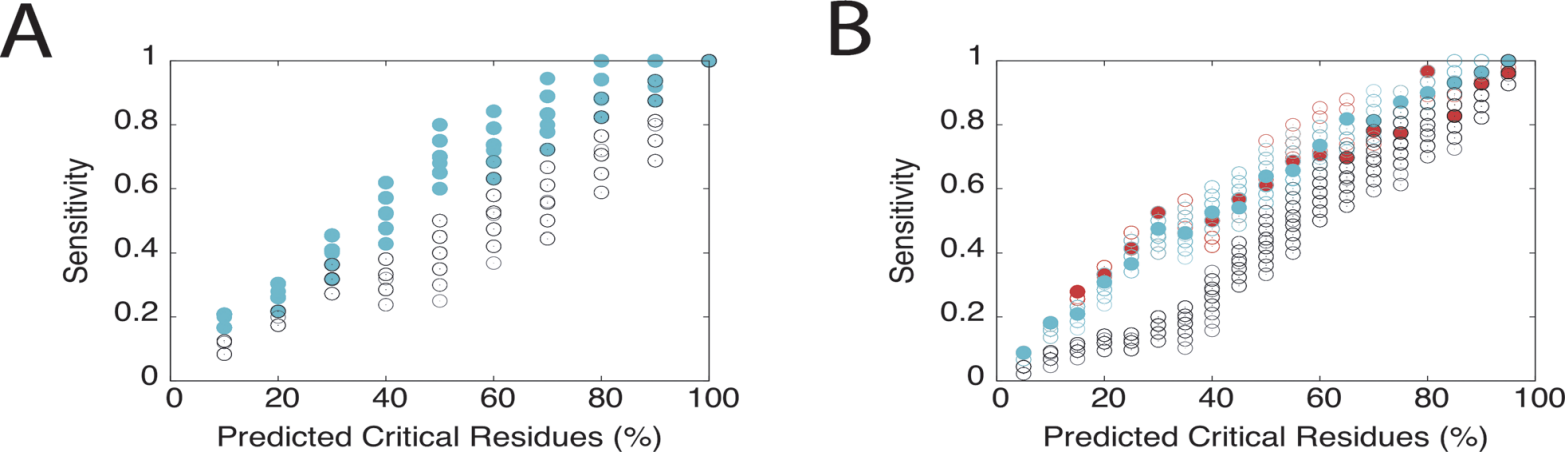
Effect on the sensitivity of critical residues prediction on HokC or the HIV-1 protease by considering ISPAs. A) Prediction of the known critical residues of HokC is shown for ConSurf (cyan circles) and a random predictor (black circles). B) Prediction of the known critical residues of the HIV-1 protease is shown for ConSurf (cyan circles), POOL (red circles) and a random predictor (black circles). The filled circles correspond to the actual predictions achieved by any of these predictors and the empty circles correspond to the variation on the reliability of these predictions when 20% of the 25 or 46 known critical residues of HokC or the HIV-1 protease are considered ISPAs, respectively. Plots show in the Y-axis the sensitivity values. The image was generated using gnuplot.

To estimate the effect on critical residue predictions on HokC by the presence of incorrect critical residue assignments, we used a predictor based on sequence conservation and a perfect predictor; for comparison, we also analysed the effect on the accuracy of these predictors on the HIV-1 protease where a predictor based on the three-dimensional structure of proteins was added to our analysis (see Methods). Assuming 20% of ISPAs in HokC and the HIV-1 protease, the reliability of these predictors was estimated (*i*.*e*., sensitivity, specificity and Matthews Correlation Coefficient) by generating 100 different sets of new critical residues for these proteins (see Figs. 4 and 5).

Note that the region in the X-axis of Fig. 4 (% of residues in a protein to be considered critical by a predictor) where the perfect predictor and a random predictor do not overlap identifies the *reliable region of a predictor*; this region is approximately equal for all three statistical parameters evaluated here; hence, we will only describe the effect on the sensitivity for simplicity. If such reliable region includes the percentage of known critical residues, we say that the predictor is *competent* to reproduce the nature of the critical residues. As expected, the presence of ISPAs did not have an impact on the reliability to identify all the known critical residues for a perfect prediction method (see Fig. 4A, 4B and 4C). On the contrary, for non-perfect predictors it is expected that the presence of ISPAs may reduce the reliable region. Indeed, the reliability of any predictor tested here was affected by altering 20% of the known critical residues in HokC (see Fig. 5A) or the HIV-1 protease (see Fig. 5B).

Next, we explored the effect on the competence of these predictors by the presence of ISPAs. In the case of HokC, 50% of the residues have been identified to have a critical role for their function (see Methods); coincidently, the reliable region for a sequence-based predictor on HokC extends up to 50% of the residues of the proteins. Thus, all the critical residues identified in HokC by our procedure have some degree of sequence conservation (see Table A in S3 Data). For comparison we used the results reported for the HIV-1 protease where critical residues were identified in the absence of a lethal condition. In such case, assuming 20% of ISPAs rendered a reliable region for a sequence-based predictor that extends up to 45% of the residues of the protein (see Fig. 5B); yet, for the predictor based on three-dimensional structure this reliable region extends up to 50% the protein residues. For the HIV-1 protease, 46 out of 99 (46%) have been identified as critical (see Methods), thus the competence of the sequence-based predictor to reproduce the nature of the structure-function relationship for the HIV-1 protease is affected when 20% of the critical residues are assumed to be incorrect. Interestingly, the authors of that mutagenesis experiment proposed that critical residues of HIV-1 protease should be those that upon mutation by a non-conservative amino acid may loose protease activity [15]. Under that criterion, 23 out of the 46 critical residues found in the HIV-1 protease would be considered critical (see Methods). This percentage of critical residues (23%) falls within the reliable region of predictors based on both sequence and the three-dimensional structure of the protease (see Fig. 5B). Not surprisingly, using a sequence conservation criterion to define critical residues as in the case of HIV-1 protease induces a bias in the list of critical residues that match the criteria used by the sequence conservation predictor, yet such criterion ignores other 23 residues that upon a conservative mutations loose protease activity.

## Discussion

Here we show that after a round of mutagenesis by PCR of *hokC*, 24 single-point mutations were identified that affect the killing activity of this gene, including 6 mutations on fully conserved residues (see Fig. 2 and Table A in S3 Data). Of particular interest is the observation that mutations on the N-terminus of HokC did have an effect on its toxic activity. These results do not agree with previous results that suggested that only the C-terminus end of HokC is relevant for its activity [21]; furthermore, many critical residues identified in this screening are located in non-conserved positions (see Table A in S3 Data). These results suggest that some of these newly identified critical residues for HokC might not be correct. Using our method CHISPAs we identified that 12–20% of the assignment in our experiment are true ISPAs (depending on the chosen statistical model to filter out false ISPAs), a frequency below the previously reported frequencies of ISPAs in saturation mutagenesis experiments [8,9,10]; this low rate of ISPAs may be explained by our strategy to sequence DNA samples extracted from cells that were not exposed to a lethal condition (see Results) and more importantly, because these previous studies reporting ISPAs did not filter out false ISPAs. Thus, CHISPAs may reduce the frequency of ISPAs in large mutagenesis experiments.

An important aspect of CHISPAs is that correcting for the ISPAs might have an impact on the reliability of critical residues predictors. To test for this idea, we simulated a perfect predictor and assumed 20% of ISPAs in HokC (p<0.01). We found that correcting the list of critical residues by considering ISPAs does alter the predictor performance even at rates as low as 20% of ISPAs; a smaller effect on the reliability of these predictors is observed when considering smaller ISPAs frequencies (data not shown). Thus, predictors that have been tested with critical residues that include false ISPAs may have larger effects on their reliability. Furthermore, our results indicate that even at low frequencies of ISPAs (*e*.*g*., 20%) the reliability and competence of predictors of critical residues is affected; these effects are more pronounced for those methods based on protein sequence than for those based on protein structure.

To further validate the ISPAs identified by our procedure, we analysed the phenotype assignments for the wild-type sequence (Ile16, Thr17 and Ala18) of *hokC* (see Table D in S3 Data). We noted that the wild-type sequence was found 510 times assigned to the mutant phenotype, which corresponds to a frequency (28%) larger than expected from experimental errors assuming any one of the statistical models; thus, assigning the wild-type sequence to a mutant phenotype clearly corresponds to an ISPA. Furthermore, the most frequent amino acid substitution among the true ISPAs was Proline (see Table D in S3 Data). These results indicate that substitutions for Proline residue may be tolerated in this transmembrane region; in agreement with this observation, previous mutagenesis experiments showed that in some transmembrane regions, Prolines can be accommodated to play a role for helix packing and signal transduction [22]. Thus, CHISPAs may be used to further test the relevance of critical residues in transmembrane proteins.

We envision some possible adaptations to our method. For instance, it is possible to sequence the DNA library of mutants before transforming cells and use this as a control distribution. Yet, without considering the experimental errors (*E+G*) such control distribution will not necessarily identify ISPAs but enriched mutations in the selection and sequencing procedures; such enrichment may assist to identify ISPAs resulting from DNA amplification if combined with our procedure. Another possible modification to our procedure is to use different statistical methods to model the sequence data; these models have to consider the binomial nature of the data, the simplicity of the model and the time-efficiency of the test. In this work we present two possible binomial models that have their own strengths and weakness. For instance, generalized linear models are commonly used in the analysis of biological data [23] because these offer many options to model biological data, yet it is not always trivial to choose the correct linear model [24,25]; here we propose the use of Bayes approximation to model the expected error distribution, but this approach requires several parameter estimations that are computationally expensive; for instance, it required about 8 hours in a 400Mhz PC with 768MB RAM running Microsoft Windows NT to perform Bayesian analyses of the data from this experiment. The results presented here may be used to further test alternative statistical analysis to check for ISPAs.

In summary, we present a method aimed to identify false relationships between DNA mutations and a biological trait by using high-throughput DNA sequencers and quantitative comparison with expected rates of experimental errors. We also show how the results from this approach may be used to assess our understanding on the structure-function relation of proteins.

## Supporting Information

S1 Data Sensitivity, specificity and MCC scores for HokC critical residues predictions. The data included in this file correspond to the values reported in Fig. 4A, 4B and 4C of this work. These include the sensitivity (Fig. 4A), specificity (Fig. 4B) and Mathews Correlation Coefficient (Fig. 4C) scores obtained by perfect and random predictors of the critical residues of HokC.
(XLS)

S2 Data Sensitivity scores for HokC and HIV1 protease critical residues predictions. The data included in this file correspond to the values reported in Fig. 5A and 5B of this work. These include the sensitivity scores obtained by a random and ConSurf predictors of the critical residues of HokC (Fig. 5A) and the HIV1 protease (Fig. 5B); for this last protein, the sensitivity scores for the POOL predictor are also reported.
(XLS)

S3 Data Supporting tables. Table A, Conserved and reported critical residues for HokC. The table shows a multiple sequence alignment obtained from PFAM database [26] for *hokC* and its homologues. From this, the conserved residues are marked on top of the aligment (*) as well as the reported residues known to be critical for *hokC* function [20] (labeled as “Reported Mutations”). Table B, Amino acid variants at the central transmembrane region of HokC isolated from colonies with wild-type phenotype. Frequency of occurrence of every sequence observed in the screening of the mutagenesis performed on the TM region of *hokC* presenting a wild type phenotype. The wild-type sequence is indicated in bold. Table C, Amino acid variants at the central transmembrane region of HokC isolated from colonies with mutant phenotype. Frequency of occurrence of every sequence observed in the screening of the mutagenesis performed on the TM region of *hokC* presenting a mutant phenotype. The wild-type sequence is indicated in bold. Table D, Amino acid variants at the central transmembrane region of HokC isolated from colonies with both mutant and wild-type phenotypes. Frequency of occurrence of every sequence observed in the screening of the mutagenesis performed on the TM region of *hokC* presenting both wild type (WT) and mutant (Mutant) phenotypes. From these frequencies, the observed incorrect sequence-phenotype assignments rates (100*Mutant Frequency/[WT Frequency + Mutant Frequency]) is shown in the table in the column labeled “Observed ISPAs (%)”. The expected rate values according to two statistical methods (BiM and ByM, see Methods) are indicated in the columns labeled “BiM Z-score (α = 5%)” and “ByM Mean”, “ByM HPD lower limit” or “ByM HPD upper limit”; in the ByM columns; HPD stands for Highest Posterior Density. The data for the wild-type sequence are highlighted in bold. For the BiM any sequence with Z-score < = 1.65 or in the case of the ByM any positive mean value has a frequency of ISPAs that may be explained by the expected rate of error from experimental/technical reasons with 95% of confidence.
(DOC)
