# Supplementary material for: Quality Control Test for Sequence-Phenotype Assignments
Source: PLoS One. 2015 Feb 20;10(2):e0118288. doi: 10.1371/journal.pone.0118288 (PMC4336291; doi:10.1371/journal.pone.0118288)
Supplement: S3 Data — Table A, Conserved and reported critical residues for HokC. The table shows a multiple sequence alignment obtained from PFAM database [26] for hokC and its homologues. From this, the conserved residues are marked on top of the aligment (*) as well as the reported residues known to be critical for hokC function [20] (labeled as “Reported Mutations”). Table B, Amino acid variants at the central transmembrane region of HokC isolated from colonies with wild-type phenotype. Frequency of occurrence of every sequence observed in the screening of the mutagenesis performed on the TM region of hokC presenting a wild type phenotype. The wild-type sequence is indicated in bold. Table C, Amino acid variants at the central transmembrane region of HokC isolated from colonies with mutant phenotype. Frequency of occurrence of every sequence observed in the screening of the mutagenesis performed on the TM region of hokC presenting a mutant phenotype. The wild-type sequence is indicated in bold. Table D, Amino acid variants at the central transmembrane region of HokC isolated from colonies with both mutant and wild-type phenotypes. Frequency of occurrence of every sequence observed in the screening of the mutagenesis performed on the TM region of hokC presenting both wild type (WT) and mutant (Mutant) phenotypes. From these frequencies, the observed incorrect sequence-phenotype assignments rates (100*Mutant Frequency/[WT Frequency + Mutant Frequency]) is shown in the table in the column labeled “Observed ISPAs (%)”. The expected rate values according to two statistical methods (BiM and ByM, see Methods) are indicated in the columns labeled “BiM Z-score (α = 5%)” and “ByM Mean”, “ByM HPD lower limit” or “ByM HPD upper limit”; in the ByM columns; HPD stands for Highest Posterior Density. The data for the wild-type sequence are highlighted in bold. For the BiM any sequence with Z-score < = 1.65 or in the case of the ByM any positive mean value has a frequency of ISPAs that may be explain [file pone.0118288.s003.doc]

**Supplemental Material for**

**Quality Control Test for DNA Sequence-Phenotype Assignments**

Maria Teresa Lara Ortiz, Pablo Benjamín Leon Rosario, Pablo Luna-Nevarez, Alba Savin Gamez, Ana Martínez Del Campo, Gabriel Del Rio.

Instituto de Fisiologia Celular, UNAM, México

This material includes:

4 Supplemental Tables: A, B, C and D.

References Poulsen *et al*, 1989 and Punta *et al*, 2012 are included in the main document of this material.

**Table A. Conserved and reported critical residues for HokC*.***

| **Reported Mutations** | **E S K GP A F** |
| --- | --- |
| **Conserved** | *** * * * * * * **** |
| **HOKC_ECOLI/2-50** | **KQHKAMIVALIVICITAVVAALVTRKDLCEVHIRT.......GQTEVAVFTAYESE-** |
| A4SUJ6_AERS4/71-120 | MFGKTAVVSLLIVCITALGVISLVRDSLCELEVHQ.......GETEIRLNLAYEAKR |
| A4SUP6_AERS4/1-50 | MPGKTAVMALLIVCVTVLAFTALVRDSLCELSVRQ.......GGTEIRAYLAYEPRE |
| Q8X9T6_ECO57/1-46 | MPQKTIIVGML--CLTMLLTVWVLHASPCEFRVSF.......MWSEIAAFLQCKP-- |
| A7MEL1_ENTS8/3-52 | MKDNTFIWCIIIVCVTVIMFTTLSRETLCELRLRG.......AGMEIVASLACKPRE |
| Q7AHU3_ECO57/21-69 | KQHKVMIVALIVICITAVVAALVTRKDLCEVHIRT.......GQTEVAVFTAYESE- |
| Q8XA64_ECO57/21-69 | KQHKVMIVALIVXCITAVVAALVTRKDLCEVHIRT.......GQTEVAVFTAYESE- |
| Q3Z5Z9_SHISS/21-69 | KQHKAMIVALIVICITAVVAALVTRKDLCEVHIRT.......GQTEVAVFTAYESE- |
| Q93DN8_9BACT/21-69 | KQHKAMIVALIVICITAVVAALVTRKDLCEVHIRT.......GQTEVAVFTAYESE- |
| Q326K5_SHIBS/21-69 | KQHKAMIVALIVICITAVVAALVTRKDLCEVHIRT.......GQTEVAVFTAYESE- |
| HOKC_SHIFL/2-50 | KQHKAMIVALIVICITAVVAALVTRKDLCEVHIRT.......GQTEVAVFTAYESE- |
| A7ZVW5_ECOLX/21-69 | KQHKAMIVALIVICITAVVAALVTRKDLCEVHIRT.......GQTEVAVFTAYESE- |
| MOKC_ECOLI/21-69 | KQHKAMIVALIVICITAVVAALVTRKDLCEVHIRT.......GQTEVAVFTAYESE- |
| Q1RGI6_ECOUT/21-69 | KQHKAMIVALIVICITAVVAALVTRKDLCEVHIRT.......GQTEVAVFTAYESE- |
| Q32KA3_SHIDS/21-69 | KQHKAMIVALIVICITAVVAAQVTRKDLCEVHIRT.......GQTEIAVFTAYESE- |
| Q1RCG0_ECOUT/21-70 | KQQKAMLIALIVICLTVIVTALVTRKDLCEVRIRT.......GQTEVAVFTVYEPEE |
| HOKD_ECO57/2-51 | KQQKAMLIALIVICLTVIVTALVTRKDLCEVRIRT.......GQTEVAVFTAYEPEE |
| Q322Y2_SHIBS/2-51 | KQQKAMLIALIVICLTVIVTALVTRKDLCEVRIRT.......GQTEVAVFTAYEPEE |
| HOKD_SHIFL/2-51 | KQQKAMLIALIVICLTVIVTALVTRKDLCEVRIRT.......GQTEVAVFTAYEPEE |
| Q32GI0_SHIDS/2-51 | KQQKAMLIALIVICLTVIVTALVTRKDLCEVRIRT.......GQTEVAVFTAYEPEE |
| HOKD_ECOLI/2-51 | KQQKAMLIALIVICLTVIVTALVTRKDLCEVRIRT.......GQTEVAVFTAYEPEE |
| Q3Z0Z0_SHISS/2-51 | KQQKAMLIALIVICLTVIVTALVTRKDLCEVRIRT.......GQTEVAVFTAYEPEE |
| Q8X286_ECO57/21-70 | KQQKAMLIALIVICLIVIVTALVTRKDLCEVRIRT.......GQTEVAVFTAYEPEE |
| Q8X289_ECO57/21-70 | KQQKAMLIALIVICLTVIVTALVTRKDLCEVRVRT.......GQTEVAVFTAYEPEE |
| Q8X288_ECO57/21-70 | KQQKAMLIALIVICITVIVTALVTRKDLCEVRIRT.......GQTEVAVFTAYEPEE |
| Q8Z607_SALTI/2-51 | KQQKAMLIALIVICLTVIVTALVTRKDLCEVRIRT.......GQTEVAVFVDYESRE |
| Q8Z606_SALTI/21-70 | KQQKAMLIALIVICLTVIVTALVTRKDLCEVRIRT.......GQTEVAVFVDYESRE |
| Q7UD56_SHIFL/3-32 | --------------------ALVTRKDLCEVRIRT.......GQTEVAVFVDYESRK |
| Q7Y2R0_9CAUD/21-70 | KQQKAMLIALIVICLTVIVTALVTRKDLCEVRIRT.......GQTEVAVFVDYESEK |
| Q776K2_9CAUD/21-70 | KQQKAMLIALIVICLTVIVTALVTRKDLCEVRIRT.......GQTEVAVFVDYESEK |
| Q7Y371_9CAUD/21-70 | KQQKAMLIALIVICLTVIVTALVTRKDLCEVRIRT.......GQTEVAVFVDYESEK |
| Q9XJM4_BP933/21-70 | KQQKAMLIALIVICLTVIVTALVTRKDLCEVRIRT.......GQTEVAVFVDYESEK |
| Q7ADV2_ECO57/21-70 | KQQKAMLIALIVICLTVIVTALVTRKDLCEVRIRT.......GQTEVAVFVDYESEK |
| Q9T212_BP933/2-51 | KQQKAMLIALIVICLTVIVTALVTRKDLCEVRIRT.......GQTEVAVFVDYESEK |
| Q9KXA3_BPVT2/21-70 | KQQKAMLIALIVICLTVIVTALVTRKDLCEVRIRT.......GQTEVAVFVDYESEK |
| Q9KXA2_BPVT2/2-51 | KQQKAMLIALIVICLTVIVTALVTRKDLCEVRIRT.......GQTEVAVFVDYESEK |
| Q7N340_PHOLL/20-68 | MKQQKAIFIAIVICIAALAAVLVTRKDLCEVRIRS.......GQTEVAVFMDYEPR- |
| A8GIY0_9ENTR/1-40 | ----------MIICMTLIALMWITRGSLCELRIKL.......GDTEVAAILAYESKG |
| A8GAB3_9ENTR/1-50 | MQQERVVLRLMIICITLIALMWITRGSLCELRITL.......GDTEVAAILAYETEG |
| Q19NA9_ECOK1/22-71 | MPNQPIVLCILIVCLTLLIFTWLTRNSLCELRMKD.......GTREVFAILAYESGK |
| Q6MXF5_SERMA/3-52 | MPNQPIVLCILIVCLTLLIFTWLTRNSLCELRMKD.......GTREVSAILAYESGK |
| A4V9Q4_9ENTR/3-52 | LPNQPVVLCVLIVCLTLLIFTWLTRNSLCELRLKD.......GTREVSAVMDYESGK |
| Q9L5P1_SALTI/22-71 | LPNQPVVLCVLIVCLTLLIFTWLTRNSLCELRLKD.......GTREVSAVMDYESGK |
| Q935G2_SALTI/3-52 | LPNQPVVLCVLIVCLTLLIFTWLTRNSLCELRLKD.......GTREVSAVMDYESGK |
| Q47655_ECOLX/3-59 | LPRSSLVWCVLIVCLTLLIFTYLTRKSLCEIRYRDgyrevaaGYREVAAFMAYESGK |
| FLMA_ECO57/3-52 | LPRSSLVWCVLIVCLTLLIFTYLTRKSLCEIRYRD.......GYREVAAFMAYESGK |
| Q1R1U0_ECOUT/3-52 | LPRSSLVWCVLIVCLTLLIFTYLTRKSLCEIRYRD.......GYREVAAFMAYESGK |
| FLMA_ECOLI/3-52 | LPRSSLVWCVLIVCLTLLIFTYLTRKSLCEIRYRD.......GYREVAAFMAYESGK |
| A0PFL1_9GAMM/3-52 | LPRSSLVWCVLIVCLTLLIFTYLTRKSLCEIRYRD.......GHREVAAFMAYESGK |
| HOK_ECOLX/3-52 | LPRSSLVWCVLIVCLTLLIFTYLTRKSLCEIRYRD.......GHREVAAFMAYESGK |
| Q7AK77_9ZZZZ/3-52 | LPRSSLVWCVLIVCLTLLIFTYLTRKSLCEIRYRD.......GHREVAAFMAYESGK |
| Q7B3S7_ECOLX/3-52 | LPRSSLVWCVLIVCLTLLIFTYLTRKSLCEIRYRD.......GHREVAAFMAYESGK |
| Q5QJQ2_SALTY/3-52 | LPRSSLVWCVLIVCLTLLIFTYLTRKSLCEIRYRD.......GHREVAAFMAYESGK |
| A4WGQ7_9ENTR/3-52 | LPRSSLIWCVLIVCLTLLIFTYLTRKSLCEIRYKD.......GDREVAAFMAYESGK |
| A4WGP5_9ENTR/3-52 | LPRSSLIWCVLIVCLTLLIFTFLTRKSLCEIRYKD.......GDREVAAFMAYESGK |
| Q2VNZ0_ECOLX/3-52 | LPGNALIWCVLIVCCTLLIFTLLTRNRLCEVRLKD.......GYREVTATMAYESGG |
| Q9F578_ECOLX/3-52 | QPKNALTWCLLIVCCTLLIFTYLTRNRLCEVRLKD.......GDREVTASLAYESNG |
| HOKB_ECOLI/1-49 | MKHNPLVVCLLIICITILTFTLLTRQTLYELRFRD.......GDKEVAALMACTSR- |
| Q1RBZ5_ECOUT/131-163 | ---------------TILTFTL-TRQSLYELRFRD.......GDKEVAALMACTSR- |
| A7MPJ9_ENTS8/8-56 | KPLHYLLACLFMVCITILIFALMNQGTLCELTIRS.......GSQEVAAKLACT-GK |
| Q9S119_ECOLX/19-68 | FMTKYALIGVLAVCATVLCFLLIFRERLCELNIHR.......GNTVVQVTLAYEARK |
| Q0H0B2_ECOLX/1-49 | -MTKYALIGVLAVCATVLCFLLIFRERLCELNIHR.......GNTVVQVTLAYEARK |
| Q573T2_9BACT/1-44 | -MTKYTLIGLLAVCATVLCFSLIFREQLCELNIHR.......GNTVVQVTLA----- |
| Q573T3_9BACT/19-63 | FMTKYTLIGLLAVCATVLCFSLIFREQLCELNIHR.......GNTVVQVTLA----- |
| Q27TF9_ECOK1/54-103 | FMTKYALIGLLAVCATVLCFSLIFRERLCELNIHR.......GNTVVQVTLAYEARK |
| SRNB_ECOLI/19-68 | FMTKYALIGLLAVCATVLCFSLIFRERLCELNIHR.......GNTVVQVTLAYEARK |
| A7ZGU5_ECOLX/1-41 | ---------MFAVCATVLCFSLIFRERLCELNIHR.......GNTVVQVTLAYEARQ |
| A5ZT05_9FIRM/199-220 | FQKRDWIMCVIAVCVTAFYFVL-------------.......--------------- |
| Q192N9_DESHD/54-74 | FVNNYAVSSVIALCFTLLFFS--------------.......--------------- |
| Q24VV4_DESHY/54-74 | FVNNYAVSSVIALCFTLLFFS--------------.......--------------- |
| Q1ZMC7_9VIBR/16-50 | --------------ITLLVALPRVRDDLCEIQYQV.......GTQPFKAVLAFEVR- |
| HOKF_ECO57/1-50 | MLTKYALVAVIVLCLTVPGFTLLVGDSLCEFTVKE.......RNIEFRAVLAYEPKK |
| HOKF_SHIFL/1-50 | MLTKYALVAVIVLCLTVLGFTLLAGDSLCEFTVKE.......RNIEFRAVLAYEPKK |
| Q0TK90_ECOL5/13-62 | MLTKYALVAVIVLCLTVLGFTLLVGDSLCEFTVKE.......RNIEFKAVLAYEPKK |
| HOKE_SHIFL/1-50 | MLTKYALVAVIVLCLTVLGFTLLVGDSLCEFTVKE.......RNIEFKAVLAYEPKK |
| HOKE_ECO57/1-50 | MLTKYALVAVIVLCLTVLGFTLLVGDSLCEFTVKE.......RNIEFKAVLAYEPKK |
| HOKE_ECOLI/1-50 | MLTKYALAAVIVLCLTVLGFTLLVGDSLCEFTVKE.......RNIEFKAVLAYEPKK |
| A2UJ60_ECOLX/34-83 | MLTKYALAAVIVLCLTVLGFTLLVGDSLCEFTVKE.......RNIEFKAVLAYEPKK |
| A7ZXK5_ECOLX/34-83 | MLTKYALAAVIVLCLTVLGFTLLVGDSLCEFTVKE.......RNIEFKAVLAYEPKK |
| A6TD25_KLEPN/18-67 | MLTKYALVAIIVLCITVLGFTLLVHSSLCELSIKE.......RNIEFKAVLAYESKK |
| Q1R7T7_ECOUT/1-50 | MLTKYALVAIIVLCCTVLGFTLMVGDSLCELSIRE.......RGMEFKAVLAYESKK |
| HOKG_ECO57/1-50 | MLTKYALVAIIVLCCTVLGFTLMVGDSLCELSIRE.......RGMEFKAVLAYESKK |
| Q0W5R4_UNCMA/1-26 | MKTSVIVPAVLVICIVIVSAAYIIGQ---------.......--------------- |
| Q8R6P1_THETN/2-16 | KKTNYLLISLLVICL--------------------.......--------------- |
| Q8D675_VIBVU/17-65 | MPKKTALAALVVVCITAVILTALYTGSLCDIRYKD.......QQNDLSIKLAYEVR- |
| A8GGY2_9ENTR/1-50 | MRLKHVFHSLTVVCITILIFIWMIRDSLCELTIYQ.......ENITILIRLACDVKR |
| PNDA1_ECOLX/1-50 | MPQRTFLMMLIVVCVTILCFVWMVRDSLCGFRIEQ.......GNTVLVATLAYEVKR |
| Q3ZU65_ECOLX/1-50 | MPQRTFLTMLIVVCVTILCFVWMVRDSLCGFRVEQ.......GNTVLVATLAYEVKR |
| Q9Z4C2_9ZZZZ/2-51 | MPQRTFLMMLIVICVTILCFVWMVRDSLCVLRLQQ.......GNTVLVATLAYEVKR |
| Q4FIJ1_9ENTR/2-51 | MPQRTFLMMLIVICVTILCFVWMVRDSLCVLRLQQ.......GNTVLVATLAYEVKR |
| Q79VV2_SALTY/1-50 | MPQRTFLMMLIVICVTILCFVWMVRDSLCGLRLQQ.......GNTVLVATLAYEVKR |
| PNDA2_ECOLX/1-50 | MPQRTFLMMLIVICVTILCFVWMVRDSLCGLRLQQ.......GNTVLVATLAYEVKR |
| Q7DJL5_9ZZZZ/1-50 | MPQRTFLMMLIVICVTILCFVWMVRDSLCGLRLQQ.......GNTVLVATLAYEVKR |
| A8A613_ECOLX/1-50 | MPQKYRLLSLIVICFTLLFFTWMIRDSLCELHIKQ.......ESYELAAFLACKLKE |
| HOKA_ECOLI/1-50 | MPQKYRLLSLIVICFTLLFFTWMIRDSLCELHIKQ.......ESYELAAFLACKLKE |
| Q1R538_ECOUT/21-70 | MPQKYRLLSLIVICFTLLFFTWMIRDSLCELHIKQ.......ESYELAAFLACKLKE |
| Q0TBP5_ECOL5/21-70 | MPQKYRLLSLIVICFTLLFFTWMIRDSLCELHIKQ.......ESYELAAFLACKLKE |
| Q3YVT9_SHISS/1-50 | MPQKYRLLSLIVICFTLLFFTWMIRDSLCELHIKQ.......ESYELAAFLACNLKE |
| A7ZTA4_ECOLX/1-50 | MPQKYRLLSLIVICFTLLFFTWMIRDSLCELHIKQ.......GSYELAAFLACNLKE |
| Q8X2A9_ECO57/21-70 | MPQKYRLLSLIVICFTLLFFTWMIRDSLCELHIKQ.......GSYELAAFLACNLKE |
| Q328L8_SHIDS/1-50 | MPQKYRLLSLIVICFTLLFFTWMIRDSLCELHIEQ.......GGYELAAFLACNLKE |
| Q31V67_SHIBS/1-50 | MPQKYGLLSLIVICFTLLFFTWMVRDSLCELHIKQ.......GRYELAAFLACNLKE |
| A6TFH3_KLEPN/21-70 | MPQKYLLFGLVVICFTILLLTWMVRDSLCELQRRQ.......GNIELVAFLACDIKQ |

The table shows a multiple sequence alignment obtained from PFAM database [29] for *hokC* and its homologues. From this, the conserved residues are marked on top of the aligment (*) as well as the reported residues known to be critical for *hokC* function [20] (labeled as “Reported Mutations”).

**Table B. Amino acid variants at the central transmembrane region of HokC isolated from colonies with wild-type phenotype.**

| **Sequence** | **Frequency** |
| --- | --- |
| AAA | 559 |
| AAD | 6 |
| AAE | 1 |
| AAG | 985 |
| AAH | 1 |
| AAL | 123 |
| AAP | 4 |
| AAR | 1 |
| AAV | 5 |
| ADA | 1 |
| AEA | 823 |
| AED | 2 |
| AEH | 1 |
| AEL | 92 |
| AEP | 1 |
| AFA | 2 |
| AGA | 7 |
| AGG | 952 |
| AGI | 1 |
| AGQ | 1 |
| AGR | 3 |
| AGV | 519 |
| AHA | 1551 |
| AHD | 1057 |
| AHG | 2 |
| AHH | 137 |
| AHK | 1 |
| AHL | 487 |
| AHN | 1 |
| AHP | 1275 |
| AHQ | 572 |
| AHR | 6 |
| AHS | 1 |
| AHT | 3 |
| AHV | 2 |
| AIA | 1 |
| AIH | 2 |
| AIV | 1 |
| AKH | 1 |
| AKP | 1 |
| AKR | 2 |
| ALA | 764 |
| ALG | 2 |
| ALH | 4 |
| ALL | 1137 |
| ALP | 829 |
| ALQ | 1467 |
| ALR | 1 |
| ALV | 454 |
| ANA | 3 |
| ANR | 1 |
| APA | 3161 |
| APD | 4 |
| APE | 120 |
| APG | 14 |
| APH | 3 |
| APL | 5 |
| APP | 30 |
| APQ | 371 |
| APR | 550 |
| APT | 5 |
| APV | 9 |
| AQA | 589 |
| AQE | 1 |
| AQG | 780 |
| AQH | 3 |
| AQP | 13 |
| AQQ | 1254 |
| AQR | 3 |
| AQV | 5 |
| ARA | 704 |
| ARD | 1 |
| ARE | 2 |
| ARG | 5 |
| ARL | 118 |
| ARP | 6 |
| ARR | 6 |
| ART | 1 |
| ARV | 462 |
| ASA | 2 |
| ASG | 1 |
| ASR | 1 |
| ATR | 1 |
| AVA | 2106 |
| AVD | 28 |
| AVE | 647 |
| AVG | 4 |
| AVH | 2012 |
| AVL | 376 |
| AVP | 5 |
| AVR | 435 |
| AVV | 8 |
| AYL | 1 |
| CGG | 1 |
| CLP | 227 |
| CLV | 1 |
| CVG | 2 |
| DAA | 885 |
| DAE | 3 |
| DAP | 2 |
| DAT | 2 |
| DAV | 5 |
| DEC | 158 |
| DEF | 1 |
| DEG | 2 |
| DER | 1 |
| DHP | 1 |
| DHR | 1 |
| DLD | 1 |
| DLP | 2 |
| DLR | 10 |
| DPH | 1 |
| DPP | 8 |
| DPR | 1 |
| DRG | 1 |
| DRH | 26 |
| DTQ | 1 |
| DVA | 2 |
| DVG | 6 |
| DVL | 1 |
| EAA | 1245 |
| EAD | 1 |
| EAH | 1 |
| EAL | 1 |
| EAP | 2 |
| EAR | 2 |
| EAS | 1 |
| EAT | 1 |
| EAV | 1 |
| EEA | 1 |
| EGA | 5 |
| EGL | 1 |
| EHA | 3 |
| EHG | 3 |
| ELA | 1 |
| ELP | 2 |
| ELQ | 2 |
| EPA | 315 |
| EPG | 3 |
| EPL | 1 |
| EPP | 4 |
| EPQ | 1 |
| EPR | 2 |
| EQD | 3 |
| ERG | 3 |
| ETA | 3 |
| ETL | 1 |
| EVA | 635 |
| EVD | 2 |
| EVF | 2 |
| EVG | 1 |
| EVH | 997 |
| EVL | 893 |
| EVP | 5 |
| EVR | 2 |
| EVV | 4 |
| EVY | 1 |
| FGG | 1 |
| FGL | 1 |
| FKR | 1 |
| FLA | 1 |
| FLG | 1 |
| FPH | 1 |
| FQP | 1 |
| FRA | 3 |
| FRD | 4 |
| FRG | 1487 |
| FRV | 5 |
| FSI | 2 |
| FSL | 1 |
| GAA | 967 |
| GAC | 1 |
| GAD | 1 |
| GAG | 4 |
| GAL | 3 |
| GAP | 237 |
| GAR | 771 |
| GAV | 1 |
| GDA | 1 |
| GEG | 6 |
| GEP | 1 |
| GET | 1 |
| GGA | 262 |
| GGE | 1 |
| GGG | 155 |
| GGI | 1 |
| GGK | 112 |
| GGL | 23 |
| GGM | 970 |
| GGP | 4 |
| GGQ | 1 |
| GGV | 9 |
| GHA | 2 |
| GHD | 69 |
| GHH | 2 |
| GHR | 1 |
| GKR | 1 |
| GLA | 153 |
| GLF | 1 |
| GLG | 2 |
| GLL | 882 |
| GLP | 6 |
| GLQ | 2 |
| GLR | 203 |
| GLV | 2 |
| GMG | 4 |
| GML | 2 |
| GPA | 282 |
| GPD | 1 |
| GPG | 3 |
| GPH | 4 |
| GPL | 1 |
| GPP | 1183 |
| GPR | 6 |
| GPS | 1 |
| GPV | 1 |
| GQG | 2 |
| GQH | 3 |
| GQP | 2 |
| GQQ | 1 |
| GRA | 2 |
| GRE | 3 |
| GRG | 4 |
| GRH | 2 |
| GRM | 1 |
| GRP | 2 |
| GRQ | 3 |
| GTR | 1 |
| GVA | 956 |
| GVD | 4 |
| GVE | 1 |
| GVG | 5775 |
| GVH | 2 |
| GVL | 818 |
| GVP | 8 |
| GVR | 3 |
| GVV | 9 |
| HAA | 2 |
| HAG | 1512 |
| HAH | 1 |
| HAL | 7 |
| HAP | 2 |
| HAS | 1 |
| HAV | 4 |
| HCL | 2 |
| HCV | 1 |
| HFA | 2 |
| HFH | 1 |
| HGC | 1 |
| HGG | 1 |
| HGP | 17 |
| HGQ | 1 |
| HGV | 1 |
| HHH | 265 |
| HHL | 2 |
| HHQ | 1 |
| HHR | 6 |
| HHV | 1 |
| HLA | 292 |
| HLD | 1 |
| HLH | 2833 |
| HLL | 191 |
| HLP | 101 |
| HLR | 722 |
| HLV | 2 |
| HPA | 10 |
| HPD | 544 |
| HPE | 1 |
| HPG | 2 |
| HPH | 95 |
| HPL | 7 |
| HPP | 5 |
| HPQ | 4 |
| HPR | 4 |
| HPV | 115 |
| HQG | 2 |
| HQH | 1 |
| HQL | 714 |
| HQP | 2 |
| HRA | 7 |
| HRH | 1 |
| HRL | 1388 |
| HRP | 5 |
| HRQ | 1 |
| HRR | 2 |
| HRV | 768 |
| HSG | 14 |
| HSP | 1 |
| HTH | 2 |
| HTL | 2 |
| HTP | 4 |
| HTQ | 1258 |
| HTR | 1 |
| HVD | 2 |
| HVG | 4 |
| HVV | 3 |
| IAA | 1 |
| IAG | 1 |
| IAL | 1 |
| IAP | 1 |
| IGA | 903 |
| IGS | 4 |
| IIP | 2 |
| IKG | 1 |
| ILL | 2 |
| ILV | 1 |
| INP | 3 |
| IPA | 3 |
| IPP | 2 |
| IPV | 2 |
| IQP | 1 |
| IRP | 2 |
| IRV | 1 |
| ISG | 1 |
| ISP | 1 |
| **ITA** | **1288** |
| ITG | 1 |
| ITH | 3 |
| ITL | 3 |
| ITP | 1730 |
| ITS | 2 |
| ITT | 1 |
| IVA | 1 |
| KER | 2 |
| KHV | 1 |
| KKA | 2 |
| KKG | 4 |
| KKV | 385 |
| KLL | 1 |
| KLV | 1 |
| KPA | 1 |
| KRG | 1 |
| KSG | 2 |
| KTV | 1 |
| KVH | 1 |
| LAA | 158 |
| LAG | 33 |
| LAH | 4 |
| LAL | 2 |
| LAP | 2043 |
| LAR | 1 |
| LAS | 1 |
| LAT | 1 |
| LAV | 3 |
| LCG | 1 |
| LCV | 1 |
| LDL | 1 |
| LDP | 1260 |
| LDR | 1 |
| LDS | 1 |
| LEL | 1 |
| LEP | 1771 |
| LES | 5 |
| LFP | 1 |
| LGA | 293 |
| LGD | 1 |
| LGE | 2 |
| LGG | 2 |
| LGH | 393 |
| LGI | 3 |
| LGL | 866 |
| LGP | 318 |
| LGQ | 1 |
| LGV | 811 |
| LHA | 2 |
| LHC | 1 |
| LHD | 1 |
| LHG | 1 |
| LHH | 3 |
| LHL | 4 |
| LHP | 213 |
| LHQ | 57 |
| LHR | 707 |
| LHV | 1 |
| LIP | 1 |
| LKG | 15 |
| LKP | 1 |
| LLA | 3180 |
| LLD | 4 |
| LLG | 3 |
| LLH | 3 |
| LLL | 21 |
| LLP | 1437 |
| LLQ | 3 |
| LLR | 28 |
| LLS | 2 |
| LLT | 3 |
| LLV | 638 |
| LPA | 1111 |
| LPD | 1520 |
| LPE | 1 |
| LPG | 5 |
| LPH | 220 |
| LPI | 1 |
| LPL | 1174 |
| LPN | 1 |
| LPP | 32 |
| LPQ | 1320 |
| LPR | 685 |
| LPV | 2009 |
| LQA | 401 |
| LQG | 1 |
| LQH | 1 |
| LQL | 5 |
| LQP | 646 |
| LQR | 521 |
| LRA | 4199 |
| LRD | 1 |
| LRG | 844 |
| LRH | 292 |
| LRL | 2 |
| LRP | 3381 |
| LRR | 10 |
| LRS | 1 |
| LRT | 6 |
| LRV | 1377 |
| LRY | 1 |
| LSA | 1 |
| LSG | 1 |
| LSL | 2 |
| LSP | 1 |
| LSV | 6 |
| LTA | 11 |
| LTP | 11 |
| LTR | 1 |
| LTV | 1 |
| LVA | 422 |
| LVD | 1 |
| LVL | 18 |
| LVP | 6 |
| LVR | 306 |
| LVT | 1 |
| LVV | 2 |
| LYG | 1 |
| MAA | 7 |
| MEG | 1 |
| MEP | 1 |
| MIA | 1 |
| MKA | 1 |
| MKD | 2 |
| MKG | 1674 |
| MKV | 4 |
| MLL | 1904 |
| MLS | 2 |
| MLV | 1 |
| MLW | 1 |
| MPG | 1 |
| MQG | 3 |
| MRG | 5 |
| MRR | 2 |
| MTG | 2 |
| NLH | 2 |
| NLL | 1 |
| NNG | 1 |
| NPG | 1 |
| NPP | 1 |
| NRG | 4 |
| NSA | 7 |
| NSC | 1 |
| NSD | 4 |
| NSG | 2555 |
| NSV | 3 |
| PAA | 176 |
| PAD | 2 |
| PAE | 7 |
| PAG | 42 |
| PAH | 2 |
| PAL | 485 |
| PAP | 13 |
| PAQ | 9 |
| PAR | 5 |
| PAV | 3375 |
| PDG | 2 |
| PDP | 3 |
| PEA | 2 |
| PEE | 2 |
| PEP | 1 |
| PEQ | 5 |
| PER | 3 |
| PEV | 2 |
| PFV | 1 |
| PGA | 6 |
| PGE | 1 |
| PGG | 5 |
| PGH | 3 |
| PGL | 1 |
| PGP | 7 |
| PGQ | 2 |
| PGR | 4 |
| PGV | 4 |
| PHA | 10 |
| PHC | 1 |
| PHD | 5 |
| PHF | 1 |
| PHG | 6 |
| PHH | 8 |
| PHL | 8 |
| PHP | 563 |
| PHQ | 1 |
| PHR | 1082 |
| PIG | 1 |
| PIR | 1 |
| PIV | 2 |
| PKH | 1 |
| PKQ | 1 |
| PKR | 1 |
| PLA | 2825 |
| PLD | 4 |
| PLE | 2 |
| PLF | 1 |
| PLG | 4 |
| PLH | 4 |
| PLL | 654 |
| PLM | 1 |
| PLP | 50 |
| PLQ | 3 |
| PLR | 6 |
| PLT | 1 |
| PLV | 1346 |
| PMV | 1 |
| PPA | 586 |
| PPD | 3 |
| PPE | 3 |
| PPG | 140 |
| PPH | 53 |
| PPL | 28 |
| PPP | 1307 |
| PPQ | 1222 |
| PPR | 18 |
| PPS | 1 |
| PPT | 1 |
| PPV | 27 |
| PQA | 6 |
| PQC | 1 |
| PQF | 3 |
| PQG | 3 |
| PQH | 6 |
| PQL | 1036 |
| PQP | 17 |
| PQQ | 2 |
| PQR | 1947 |
| PQV | 305 |
| PRA | 14 |
| PRD | 3 |
| PRG | 4 |
| PRH | 2 |
| PRL | 12 |
| PRP | 65 |
| PRQ | 3 |
| PRR | 6 |
| PRV | 734 |
| PSG | 5 |
| PSP | 281 |
| PTP | 3 |
| PTV | 1 |
| PVA | 4 |
| PVE | 2 |
| PVG | 4 |
| PVH | 7 |
| PVI | 1 |
| PVL | 1098 |
| PVM | 1 |
| PVP | 8 |
| PVQ | 2 |
| PVR | 943 |
| PVV | 1868 |
| QAA | 459 |
| QAD | 2 |
| QAG | 2 |
| QAH | 1194 |
| QAL | 277 |
| QAP | 6 |
| QAR | 1 |
| QAV | 2 |
| QDA | 1 |
| QFA | 190 |
| QGQ | 1 |
| QHA | 956 |
| QHD | 2 |
| QHH | 1 |
| QHL | 993 |
| QHP | 860 |
| QHR | 2 |
| QHT | 4 |
| QHV | 786 |
| QIA | 1 |
| QKV | 5 |
| QLA | 4 |
| QLF | 1 |
| QLG | 3 |
| QLH | 1 |
| QLI | 1 |
| QLL | 2244 |
| QLM | 1 |
| QLP | 6 |
| QLV | 2104 |
| QNL | 1 |
| QPA | 8 |
| QPG | 1224 |
| QPH | 4 |
| QPL | 12 |
| QPP | 2 |
| QPR | 6 |
| QPV | 5 |
| QQA | 1 |
| QQF | 3 |
| QQH | 3 |
| QQL | 4137 |
| QQP | 5 |
| QQR | 6 |
| QQV | 3 |
| QRA | 6 |
| QRR | 1 |
| QSH | 2 |
| QTH | 1 |
| QVA | 236 |
| QVH | 1 |
| QVL | 1 |
| QVV | 1 |
| QYL | 1 |
| QYP | 1 |
| RAA | 830 |
| RAD | 2 |
| RAG | 1 |
| RAH | 2 |
| RAL | 2 |
| RAQ | 1 |
| RAR | 1 |
| RAV | 2 |
| RDA | 2 |
| RDR | 1 |
| REA | 1 |
| REH | 1 |
| REP | 3 |
| REV | 1 |
| RGA | 1 |
| RGG | 2 |
| RGL | 5 |
| RGM | 377 |
| RGP | 1 |
| RGV | 2 |
| RHA | 4 |
| RHL | 1 |
| RHP | 1 |
| RHR | 1 |
| RIV | 2 |
| RKI | 1 |
| RLA | 1141 |
| RLD | 1 |
| RLF | 1 |
| RLG | 5 |
| RLH | 2 |
| RLL | 3 |
| RLP | 12 |
| RLQ | 1 |
| RLR | 4 |
| RLV | 1229 |
| RPA | 3 |
| RPD | 1 |
| RPE | 1 |
| RPG | 2 |
| RPH | 463 |
| RPL | 2 |
| RPP | 8 |
| RPQ | 303 |
| RPR | 3 |
| RPS | 6 |
| RPV | 2 |
| RQG | 1 |
| RQL | 4 |
| RQP | 7 |
| RRH | 1 |
| RRL | 1 |
| RRV | 1 |
| RSA | 1 |
| RTA | 1 |
| RTH | 1 |
| RTP | 1 |
| RTQ | 1 |
| RVA | 9 |
| RVD | 987 |
| RVE | 2 |
| RVG | 1 |
| RVH | 2 |
| RVL | 635 |
| RVP | 2 |
| RVQ | 1 |
| RVV | 1093 |
| RWH | 1 |
| RWM | 1 |
| SAG | 3 |
| SAR | 1 |
| SAV | 3 |
| SEA | 1 |
| SEL | 1 |
| SHA | 1 |
| SHD | 2 |
| SHL | 1 |
| SHQ | 1 |
| SKH | 534 |
| SKL | 1 |
| SLA | 1 |
| SLG | 1 |
| SLL | 1 |
| SLP | 1 |
| SLQ | 1 |
| SNH | 1 |
| SPA | 2 |
| SPQ | 1 |
| SPV | 8 |
| SQQ | 1 |
| SRH | 1 |
| SSA | 9 |
| SSD | 1 |
| SSI | 2 |
| SSL | 1 |
| SSV | 1471 |
| STH | 1 |
| SVA | 1 |
| SVD | 1 |
| SVL | 1 |
| SVV | 1 |
| SWA | 1 |
| SWL | 1 |
| SWR | 1 |
| TAG | 2 |
| TAL | 1 |
| TAV | 2 |
| TCG | 2 |
| TGV | 1 |
| THA | 1 |
| THD | 1 |
| THP | 3 |
| TIG | 1 |
| TLA | 1 |
| TLP | 1 |
| TPA | 4 |
| TPP | 1 |
| TQA | 2 |
| TQG | 1 |
| TQQ | 1 |
| TRV | 2 |
| TSG | 963 |
| TTA | 1 |
| TTG | 1 |
| TTP | 1 |
| TVH | 5 |
| TVR | 1 |
| VAA | 4 |
| VAG | 8 |
| VAH | 22 |
| VAL | 1 |
| VAP | 3 |
| VAR | 2 |
| VCG | 3 |
| VDR | 51 |
| VEA | 2 |
| VEL | 1 |
| VFQ | 1 |
| VFV | 1 |
| VGA | 1 |
| VGG | 7 |
| VGK | 1 |
| VGM | 2 |
| VGP | 1 |
| VGR | 246 |
| VGV | 400 |
| VHA | 3 |
| VHC | 1 |
| VHD | 3 |
| VHG | 1 |
| VHL | 99 |
| VHP | 1 |
| VHQ | 4 |
| VHR | 615 |
| VKH | 3 |
| VKP | 5 |
| VKR | 2091 |
| VLA | 2 |
| VLL | 29 |
| VLP | 5 |
| VLQ | 1588 |
| VLR | 4 |
| VLT | 1 |
| VLV | 1232 |
| VMR | 1 |
| VNR | 2 |
| VPA | 1831 |
| VPD | 2 |
| VPG | 274 |
| VPH | 2 |
| VPL | 1 |
| VPP | 16 |
| VPQ | 1 |
| VPR | 1 |
| VPS | 1 |
| VPT | 3 |
| VPV | 5 |
| VPY | 1 |
| VQA | 350 |
| VQG | 1 |
| VQL | 4 |
| VQP | 3489 |
| VQQ | 6 |
| VQR | 1 |
| VQT | 1 |
| VQV | 1 |
| VRA | 8 |
| VRD | 1 |
| VRE | 1 |
| VRG | 1671 |
| VRH | 1 |
| VRL | 290 |
| VRP | 9 |
| VRQ | 1 |
| VRR | 1078 |
| VRV | 4 |
| VRW | 1 |
| VTA | 2 |
| VTG | 1 |
| VTR | 1 |
| VVA | 3 |
| VVD | 2 |
| VVE | 8 |
| VVH | 11 |
| VVL | 1 |
| VVR | 67 |
| VVV | 1 |
| VWR | 1 |
| WAE | 1 |
| WEE | 3 |
| WGA | 1 |
| WGG | 1 |
| WRG | 1 |
| WRP | 1 |
| WVR | 1 |
| YAG | 1 |
| YGC | 1155 |
| YGG | 21 |
| YGR | 12 |
| YGS | 5 |
| YGY | 2 |
| YLH | 1 |
| YRL | 1 |

Frequency of occurrence of every sequence observed in the screening of the mutagenesis performed on the TM region of *hokC* presenting a wild type phenotype. The wild-type sequence is indicated in bold.

**Table C. Amino acid variants at the central transmembrane region of HokC isolated from colonies with mutant phenotype.**

| **Sequence** | **Frequency** |
| --- | --- |
| AAA | 2 |
| AAG | 686 |
| AAH | 3 |
| AAL | 2 |
| AAP | 1318 |
| AAR | 363 |
| AAV | 2 |
| ADG | 2 |
| ADP | 2 |
| AEA | 12 |
| AEG | 34 |
| AEL | 1 |
| AER | 1 |
| AGD | 1 |
| AGG | 685 |
| AGH | 3 |
| AGL | 1 |
| AGP | 660 |
| AGQ | 167 |
| AGV | 11 |
| AHA | 23 |
| AHC | 1 |
| AHD | 23 |
| AHE | 99 |
| AHG | 2 |
| AHH | 2 |
| AHL | 193 |
| AHP | 29 |
| AHQ | 223 |
| AHR | 376 |
| AIL | 1 |
| ALA | 11 |
| ALG | 95 |
| ALL | 535 |
| ALP | 233 |
| ALQ | 19 |
| ALR | 3 |
| ALS | 1 |
| ALT | 1 |
| ALV | 10 |
| APA | 1480 |
| APC | 1 |
| APD | 341 |
| APE | 219 |
| APG | 1189 |
| APH | 758 |
| API | 1 |
| APL | 686 |
| APP | 1715 |
| APQ | 1007 |
| APR | 1601 |
| APS | 2 |
| APT | 1 |
| APV | 1283 |
| AQA | 11 |
| AQD | 1 |
| AQE | 86 |
| AQG | 15 |
| AQP | 742 |
| AQQ | 11 |
| AQT | 1 |
| ARA | 483 |
| ARC | 1 |
| ARD | 211 |
| ARE | 332 |
| ARG | 465 |
| ARH | 218 |
| ARI | 1 |
| ARL | 11 |
| ARP | 2142 |
| ARQ | 2 |
| ARR | 1154 |
| ARS | 1 |
| ART | 1 |
| ARV | 1428 |
| ASP | 1 |
| ASR | 2 |
| ATP | 3 |
| ATQ | 1 |
| ATR | 1 |
| AVA | 69 |
| AVD | 1 |
| AVE | 11 |
| AVH | 28 |
| AVL | 1041 |
| AVP | 579 |
| AVR | 11 |
| AVS | 1 |
| AWP | 3 |
| CGH | 1 |
| CKV | 1 |
| CLP | 2 |
| CPG | 1 |
| CSG | 122 |
| CWH | 1 |
| DAA | 11 |
| DCG | 1 |
| DDP | 1 |
| DEC | 3 |
| DGA | 1 |
| DGP | 1 |
| DHG | 1 |
| DHP | 344 |
| DHS | 1 |
| DLP | 120 |
| DML | 2 |
| DNS | 1 |
| DPA | 1 |
| DPG | 1 |
| DPH | 459 |
| DPL | 1 |
| DPP | 722 |
| DPR | 1 |
| DQH | 2 |
| DQP | 110 |
| DRA | 1 |
| DRE | 1 |
| DRG | 429 |
| DRH | 1 |
| DSS | 345 |
| DST | 1 |
| DVL | 1 |
| DVP | 229 |
| EAA | 35 |
| EAP | 228 |
| EAQ | 1 |
| EEA | 436 |
| EEP | 1 |
| EEV | 1 |
| EGA | 907 |
| EGD | 1 |
| EGL | 135 |
| EGP | 1 |
| EGS | 1 |
| EGT | 3 |
| EGV | 3 |
| EHA | 950 |
| EHG | 622 |
| EHP | 2 |
| EHT | 1 |
| EHV | 1 |
| ELD | 1 |
| ELG | 1 |
| ELP | 219 |
| ENR | 1 |
| EPA | 214 |
| EPD | 8 |
| EPG | 435 |
| EPL | 2 |
| EPP | 666 |
| EPQ | 2 |
| EPR | 984 |
| EPV | 1 |
| EQA | 1 |
| EQD | 360 |
| ERA | 32 |
| ERD | 1 |
| ERG | 741 |
| ERP | 2 |
| ERW | 2 |
| ESA | 1 |
| ESG | 3 |
| ESR | 1 |
| EVA | 12 |
| EVD | 174 |
| EVH | 11 |
| EVL | 10 |
| EVP | 886 |
| EVQ | 1 |
| EVV | 1 |
| EYG | 1 |
| FRG | 26 |
| FSI | 732 |
| FSL | 1 |
| GAA | 101 |
| GAG | 11 |
| GAL | 185 |
| GAP | 394 |
| GAR | 15 |
| GAS | 1 |
| GAT | 5 |
| GDG | 3 |
| GDP | 1 |
| GEE | 1 |
| GEG | 620 |
| GET | 193 |
| GGA | 482 |
| GGG | 113 |
| GGH | 1 |
| GGK | 464 |
| GGM | 12 |
| GGP | 476 |
| GGR | 1 |
| GGS | 2 |
| GGT | 3 |
| GHD | 3 |
| GHG | 1 |
| GHH | 1 |
| GHP | 120 |
| GHQ | 1 |
| GHR | 64 |
| GKS | 262 |
| GLA | 2 |
| GLH | 39 |
| GLL | 15 |
| GLP | 176 |
| GLR | 5 |
| GMF | 1 |
| GMI | 1 |
| GML | 354 |
| GMP | 1 |
| GPA | 583 |
| GPC | 2 |
| GPE | 259 |
| GPG | 3 |
| GPH | 198 |
| GPL | 1 |
| GPP | 2376 |
| GPQ | 3 |
| GPR | 1351 |
| GPS | 2 |
| GPT | 1 |
| GPV | 1 |
| GQG | 1 |
| GQH | 363 |
| GQP | 1 |
| GQR | 2 |
| GRA | 136 |
| GRE | 58 |
| GRG | 2 |
| GRH | 192 |
| GRK | 1 |
| GRL | 1 |
| GRP | 369 |
| GRQ | 811 |
| GRR | 219 |
| GRV | 1 |
| GSG | 3 |
| GSR | 1 |
| GTS | 9 |
| GVA | 12 |
| GVG | 89 |
| GVI | 1 |
| GVL | 10 |
| GVP | 1202 |
| GVR | 1 |
| GVS | 332 |
| GVT | 1 |
| GVV | 1 |
| HAA | 1 |
| HAG | 17 |
| HAH | 1 |
| HAP | 307 |
| HAV | 366 |
| HCT | 1 |
| HDP | 2 |
| HGA | 74 |
| HGP | 122 |
| HGQ | 688 |
| HGR | 2 |
| HHA | 4 |
| HHG | 6 |
| HHH | 3 |
| HHL | 2 |
| HHR | 573 |
| HKL | 1 |
| HKV | 1 |
| HLA | 270 |
| HLG | 9 |
| HLH | 30 |
| HLL | 1 |
| HLP | 1005 |
| HLR | 13 |
| HLS | 1 |
| HLW | 1 |
| HPA | 2295 |
| HPD | 7 |
| HPG | 11 |
| HPH | 576 |
| HPL | 388 |
| HPP | 461 |
| HPQ | 2 |
| HPR | 527 |
| HPS | 253 |
| HPT | 4 |
| HPV | 552 |
| HQA | 2 |
| HQG | 339 |
| HQH | 1 |
| HQL | 11 |
| HQP | 504 |
| HQQ | 1 |
| HQR | 1 |
| HQS | 1 |
| HRA | 1076 |
| HRD | 1 |
| HRH | 3 |
| HRL | 794 |
| HRP | 609 |
| HRQ | 197 |
| HRR | 1 |
| HRV | 54 |
| HSA | 1 |
| HSH | 1 |
| HSL | 1 |
| HSS | 1 |
| HTP | 1 |
| HTQ | 18 |
| HVL | 43 |
| HVP | 2 |
| HWH | 1 |
| IGA | 19 |
| IPS | 1 |
| ISI | 1 |
| **ITA** | **510** |
| ITP | 33 |
| ITT | 1 |
| KKV | 8 |
| KPA | 232 |
| KPS | 2 |
| KPV | 1 |
| KSA | 1 |
| KVP | 1 |
| LAA | 3 |
| LAG | 1 |
| LAH | 1 |
| LAL | 68 |
| LAP | 69 |
| LAR | 1 |
| LAS | 1 |
| LAV | 2 |
| LCR | 1 |
| LDL | 1 |
| LDP | 16 |
| LEA | 2 |
| LEG | 3 |
| LEP | 23 |
| LGA | 5 |
| LGG | 1 |
| LGH | 4 |
| LGL | 10 |
| LGP | 6 |
| LGV | 8 |
| LHD | 1 |
| LHH | 8 |
| LHL | 2 |
| LHP | 6 |
| LHQ | 2 |
| LHR | 657 |
| LKG | 188 |
| LLA | 614 |
| LLD | 1 |
| LLL | 1 |
| LLP | 362 |
| LLQ | 166 |
| LLV | 7 |
| LPA | 98 |
| LPD | 275 |
| LPF | 8 |
| LPG | 5 |
| LPH | 5 |
| LPL | 260 |
| LPP | 2448 |
| LPQ | 13 |
| LPR | 28 |
| LPS | 2 |
| LPT | 1 |
| LPV | 171 |
| LPW | 1 |
| LQA | 8 |
| LQC | 1 |
| LQH | 1 |
| LQL | 333 |
| LQP | 8 |
| LQR | 268 |
| LRA | 70 |
| LRC | 1 |
| LRG | 237 |
| LRH | 3 |
| LRL | 1 |
| LRP | 49 |
| LRQ | 14 |
| LRR | 506 |
| LRT | 1 |
| LRV | 19 |
| LSI | 2 |
| LSL | 2 |
| LSP | 8 |
| LTG | 4 |
| LTP | 1 |
| LVA | 4 |
| LVE | 3 |
| LVP | 26 |
| LVQ | 1 |
| LVR | 2 |
| LYP | 3 |
| MKG | 26 |
| MLL | 25 |
| MLQ | 1 |
| MRP | 1 |
| NGV | 1 |
| NHP | 1 |
| NLV | 1 |
| NPH | 1 |
| NPQ | 4 |
| NPS | 1 |
| NQP | 1 |
| NQQ | 106 |
| NSG | 37 |
| NVA | 2 |
| NVE | 1 |
| NVG | 1 |
| NVM | 1 |
| NVV | 332 |
| PAA | 1267 |
| PAD | 2 |
| PAE | 25 |
| PAG | 853 |
| PAH | 933 |
| PAK | 1 |
| PAL | 580 |
| PAP | 1720 |
| PAQ | 272 |
| PAR | 824 |
| PAS | 2 |
| PAT | 1 |
| PAV | 717 |
| PCG | 2 |
| PCP | 2 |
| PDA | 3 |
| PDE | 7 |
| PDG | 16 |
| PDL | 2 |
| PDP | 295 |
| PDQ | 5 |
| PDR | 6 |
| PDV | 4 |
| PEA | 650 |
| PED | 1 |
| PEE | 1131 |
| PEG | 1116 |
| PEH | 270 |
| PEL | 378 |
| PEM | 1 |
| PEP | 841 |
| PEQ | 640 |
| PER | 472 |
| PES | 1 |
| PEV | 1 |
| PFP | 2 |
| PFR | 1 |
| PGA | 1245 |
| PGC | 1 |
| PGD | 1 |
| PGG | 1159 |
| PGH | 2 |
| PGL | 288 |
| PGP | 1543 |
| PGQ | 1168 |
| PGR | 1247 |
| PGT | 1 |
| PGV | 633 |
| PHA | 1858 |
| PHC | 3 |
| PHD | 280 |
| PHG | 612 |
| PHH | 965 |
| PHL | 884 |
| PHP | 3063 |
| PHQ | 558 |
| PHR | 1127 |
| PHS | 5 |
| PHT | 2 |
| PHV | 54 |
| PIE | 2 |
| PIQ | 1 |
| PKA | 1 |
| PKE | 1 |
| PKH | 1 |
| PKQ | 1 |
| PKR | 1 |
| PLA | 53 |
| PLD | 978 |
| PLG | 886 |
| PLH | 2 |
| PLL | 733 |
| PLN | 1 |
| PLP | 2130 |
| PLQ | 301 |
| PLR | 581 |
| PLS | 1 |
| PLV | 24 |
| PMR | 1 |
| PND | 1 |
| PPA | 5894 |
| PPD | 68 |
| PPE | 3 |
| PPF | 1 |
| PPG | 1425 |
| PPH | 620 |
| PPI | 3 |
| PPL | 1709 |
| PPP | 6410 |
| PPQ | 474 |
| PPR | 2896 |
| PPS | 4 |
| PPT | 10 |
| PPV | 3281 |
| PQA | 1241 |
| PQC | 1 |
| PQD | 1 |
| PQE | 1 |
| PQG | 370 |
| PQH | 1010 |
| PQL | 53 |
| PQP | 1981 |
| PQQ | 1 |
| PQR | 2004 |
| PQS | 3 |
| PQV | 9 |
| PRA | 1195 |
| PRD | 785 |
| PRF | 1 |
| PRG | 1110 |
| PRH | 496 |
| PRL | 2655 |
| PRP | 3020 |
| PRQ | 1034 |
| PRR | 791 |
| PRS | 2 |
| PRT | 1 |
| PRV | 16 |
| PSA | 2 |
| PSH | 1 |
| PSL | 1 |
| PSP | 10 |
| PSR | 2 |
| PSV | 3 |
| PTA | 2 |
| PTG | 3 |
| PTP | 3 |
| PTQ | 1 |
| PTR | 1 |
| PTV | 4 |
| PVA | 5 |
| PVE | 468 |
| PVG | 1 |
| PVH | 40 |
| PVL | 459 |
| PVP | 335 |
| PVQ | 877 |
| PVR | 1939 |
| PVV | 352 |
| PVW | 1 |
| PWQ | 1 |
| PYA | 1 |
| PYD | 1 |
| QAA | 9 |
| QAD | 1 |
| QAG | 142 |
| QAH | 22 |
| QAL | 6 |
| QAP | 340 |
| QAQ | 5 |
| QAV | 76 |
| QDP | 211 |
| QDQ | 1 |
| QEG | 1 |
| QEP | 1 |
| QEQ | 207 |
| QER | 66 |
| QFA | 3 |
| QGA | 88 |
| QGD | 2 |
| QGP | 5 |
| QGQ | 2 |
| QHA | 10 |
| QHC | 2 |
| QHH | 1 |
| QHL | 19 |
| QHP | 504 |
| QHR | 174 |
| QHV | 7 |
| QLA | 1 |
| QLL | 32 |
| QLP | 203 |
| QLV | 30 |
| QPA | 638 |
| QPG | 22 |
| QPH | 8 |
| QPL | 1 |
| QPP | 15 |
| QPQ | 4 |
| QPR | 955 |
| QPT | 1 |
| QQH | 315 |
| QQL | 50 |
| QQR | 1 |
| QQY | 1 |
| QRA | 1037 |
| QRE | 8 |
| QRG | 1 |
| QRP | 2 |
| QRQ | 1 |
| QRT | 1 |
| QRV | 1 |
| QVA | 4 |
| QVD | 238 |
| QVG | 1 |
| QVS | 1 |
| QWA | 1 |
| RAA | 20 |
| RAG | 73 |
| RAH | 98 |
| RAN | 1 |
| RAP | 286 |
| RAR | 5 |
| RAV | 1 |
| RDA | 324 |
| RDG | 1 |
| RDP | 67 |
| RDR | 440 |
| RDV | 1 |
| RDW | 1 |
| REE | 2 |
| REH | 514 |
| REI | 1 |
| REP | 418 |
| REQ | 1 |
| REY | 3 |
| RGA | 216 |
| RGD | 1 |
| RGE | 1 |
| RGG | 658 |
| RGH | 320 |
| RGL | 1 |
| RGM | 12 |
| RGP | 935 |
| RGQ | 1 |
| RGR | 1 |
| RGS | 1 |
| RGT | 1 |
| RHA | 1231 |
| RHG | 1 |
| RHL | 3 |
| RHP | 1 |
| RHR | 275 |
| RHT | 1 |
| RHV | 2 |
| RKA | 4 |
| RKG | 1 |
| RKI | 257 |
| RKT | 2 |
| RKV | 228 |
| RLA | 21 |
| RLC | 1 |
| RLD | 1 |
| RLG | 753 |
| RLH | 239 |
| RLL | 2 |
| RLP | 128 |
| RLQ | 544 |
| RLR | 1596 |
| RLV | 13 |
| RLY | 1 |
| RMV | 1 |
| RNA | 1 |
| RPA | 270 |
| RPG | 573 |
| RPH | 52 |
| RPL | 231 |
| RPP | 1331 |
| RPQ | 6 |
| RPR | 247 |
| RPS | 383 |
| RPT | 3 |
| RQG | 28 |
| RQI | 1 |
| RQV | 1 |
| RRA | 13 |
| RRE | 6 |
| RRG | 25 |
| RRH | 2 |
| RRL | 358 |
| RRP | 323 |
| RRQ | 1 |
| RRR | 2 |
| RRV | 436 |
| RSA | 2 |
| RSP | 1 |
| RTA | 1 |
| RTI | 2 |
| RTP | 1 |
| RTV | 4 |
| RVA | 21 |
| RVD | 21 |
| RVE | 1 |
| RVH | 161 |
| RVL | 16 |
| RVP | 302 |
| RVR | 1 |
| RVV | 26 |
| RWH | 881 |
| RWL | 1 |
| RWP | 2 |
| RWR | 1 |
| RWV | 1 |
| SAG | 190 |
| SAR | 1 |
| SAS | 2 |
| SEH | 1 |
| SGA | 69 |
| SGG | 3 |
| SGL | 1 |
| SGP | 1 |
| SGV | 1 |
| SHG | 1 |
| SKH | 2 |
| SPA | 1 |
| SPL | 1 |
| SPP | 4 |
| SPS | 1 |
| SPV | 1 |
| SQR | 1 |
| SRA | 153 |
| SRG | 1 |
| SRL | 1 |
| SRM | 2 |
| SRP | 2 |
| SRQ | 1 |
| SRV | 2 |
| SSA | 1 |
| SSP | 46 |
| SSV | 23 |
| SWA | 67 |
| TAR | 1 |
| TEE | 1 |
| TGG | 1 |
| TGR | 181 |
| THQ | 1 |
| TLP | 1 |
| TPA | 2 |
| TPD | 1 |
| TPG | 2 |
| TPH | 1 |
| TPL | 1 |
| TPP | 4 |
| TPR | 3 |
| TPV | 2 |
| TQP | 1 |
| TRD | 2 |
| TRE | 1 |
| TRG | 1 |
| TRP | 5 |
| TRR | 3 |
| TRV | 1 |
| TSG | 12 |
| TVL | 1 |
| TVP | 1 |
| VAA | 19 |
| VAE | 1 |
| VAG | 2 |
| VAL | 1 |
| VAP | 1076 |
| VAS | 33 |
| VAV | 1 |
| VDP | 367 |
| VDR | 2 |
| VEP | 1 |
| VFV | 1 |
| VGD | 10 |
| VGG | 2 |
| VGH | 1 |
| VGL | 1 |
| VGP | 205 |
| VGR | 307 |
| VGV | 8 |
| VGW | 1 |
| VHA | 2 |
| VHH | 2 |
| VHN | 1 |
| VHP | 2 |
| VHR | 353 |
| VKR | 36 |
| VLA | 2 |
| VLP | 192 |
| VLQ | 32 |
| VLV | 14 |
| VNP | 1 |
| VPA | 245 |
| VPC | 2 |
| VPD | 1 |
| VPG | 7 |
| VPH | 728 |
| VPL | 5 |
| VPM | 2 |
| VPP | 1961 |
| VPQ | 2 |
| VPR | 414 |
| VPV | 835 |
| VQA | 6 |
| VQP | 63 |
| VQV | 1 |
| VRA | 2 |
| VRD | 237 |
| VRE | 2 |
| VRG | 61 |
| VRH | 154 |
| VRL | 104 |
| VRP | 797 |
| VRQ | 1 |
| VRR | 26 |
| VRV | 3 |
| VSH | 1 |
| VTP | 2 |
| VVL | 3 |
| VVP | 4 |
| VVR | 50 |
| VVS | 1 |
| VWG | 1 |
| VWL | 2 |
| VWP | 2 |
| VWR | 1 |
| WAE | 17 |
| WEA | 4 |
| WED | 1 |
| WEE | 867 |
| WEG | 3 |
| WET | 1 |
| WGE | 2 |
| WKG | 1 |
| WLG | 1 |
| WRA | 1 |
| WRE | 296 |
| WRG | 452 |
| WRR | 1 |
| WRV | 1 |
| WVE | 1 |
| WWG | 1 |
| YGC | 18 |
| YGS | 1 |
| YHP | 1 |

Frequency of occurrence of every sequence observed in the screening of the mutagenesis performed on the TM region of *hokC* presenting a mutant phenotype. The wild-type sequence is indicated in bold.

**Table D. Amino acid variants at the central transmembrane region of HokC isolated from colonies with both mutant and wild-type phenotypes.**

| **Sequence** | **WT Frequency** | **Mutant Frequency** | **Observed ISPAs (%)** | **BiM**  **Z-score (α=5%)** | **ByM Mean** | **ByM HPD Lower limit** | **ByM HPD Upper limit** |
| --- | --- | --- | --- | --- | --- | --- | --- |
| AAA | 559 | 2 | 0.36 | -6.423779 | -7.9593 | -8.9173 | -7.04 |
| AAG | 985 | 686 | 41.05 | 52.074064 | -3.4444 | -3.7147 | -3.1663 |
| AAH | 1 | 3 | 25 | 1.328821 | -1.0469 | -3.2018 | 1.2366 |
| AAL | 123 | 2 | 1.6 | -2.504411 | -6.5452 | -7.5506 | -5.5879 |
| AAP | 4 | 1318 | 0.3 | -9.935542 | 2.6071 | 1.7532 | 3.5939 |
| AAR | 1 | 363 | 0.27 | -5.233637 | 2.4317 | 1.0688 | 3.8705 |
| AAV | 5 | 2 | 28.57 | 2.116613 | -3.323 | -4.7745 | -1.9152 |
| AEA | 823 | 12 | 1.44 | -6.651511 | -7.1566 | -7.7537 | -6.5993 |
| AEL | 92 | 1 | 1.08 | -2.352312 | -6.5374 | -7.6579 | -5.4709 |
| AGG | 952 | 685 | 41.84 | 52.757513 | -3.4121 | -3.6854 | -3.129 |
| AGQ | 1 | 167 | 0.6 | -3.397832 | 1.7989 | 0.3988 | 3.3761 |
| AGV | 519 | 11 | 2.08 | -4.741311 | -6.7751 | -7.4053 | -6.1951 |
| AHA | 1551 | 23 | 1.46 | -9.095954 | -7.2109 | -7.6785 | -6.7537 |
| AHD | 1057 | 23 | 2.13 | -6.700614 | -6.8295 | -7.3064 | -6.3864 |
| AHG | 2 | 2 | 50 | 3.227137 | -2.1507 | -4.0244 | -0.1389 |
| AHH | 137 | 2 | 1.44 | -2.71307 | -6.641 | -7.6508 | -5.6621 |
| AHL | 487 | 193 | 28.38 | 20.674375 | -4.0022 | -4.3095 | -3.6922 |
| AHP | 1275 | 29 | 2.22 | -7.233494 | -6.8019 | -7.2409 | -6.3672 |
| AHQ | 572 | 223 | 28.05 | 21.998878 | -4.0204 | -4.3345 | -3.7349 |
| AHR | 6 | 376 | 1.57 | -4.399821 | 1.0718 | 0.2788 | 1.9046 |
| ALA | 764 | 11 | 1.42 | -6.426863 | -7.1522 | -7.7398 | -6.5566 |
| ALG | 2 | 95 | 2.06 | -2.033457 | 0.8219 | -0.4371 | 2.1528 |
| ALL | 1137 | 535 | 32 | 38.031225 | -3.8351 | -4.115 | -3.5609 |
| ALP | 829 | 233 | 21.94 | 17.865699 | -4.3462 | -4.6324 | -4.0496 |
| ALQ | 1467 | 19 | 1.28 | -9.105337 | -7.3289 | -7.8105 | -6.8258 |
| ALR | 1 | 3 | 25 | 1.328821 | -1.0505 | -3.1026 | 1.3205 |
| ALV | 454 | 10 | 2.16 | -4.371105 | -6.7305 | -7.3523 | -6.1166 |
| APA | 3161 | 1480 | 31.89 | 63.082714 | -3.8428 | -4.1084 | -3.5813 |
| APD | 4 | 341 | 1.16 | -4.471333 | 1.3587 | 0.4217 | 2.3527 |
| APE | 120 | 219 | 35.4 | 19.501814 | -2.4689 | -2.8096 | -2.1257 |
| APG | 14 | 1189 | 1.16 | -8.343785 | 1.3594 | 0.7969 | 1.9394 |
| APH | 3 | 758 | 0.39 | -7.442223 | 2.3335 | 1.3156 | 3.3648 |
| APL | 5 | 686 | 0.72 | -6.762967 | 1.801 | 0.9705 | 2.677 |
| APP | 30 | 1715 | 1.72 | -9.168205 | 0.9677 | 0.541 | 1.422 |
| APQ | 371 | 1007 | 26.92 | 27.374181 | -2.084 | -2.3721 | -1.7964 |
| APR | 550 | 1601 | 25.57 | 31.817416 | -2.015 | -2.2839 | -1.7379 |
| APT | 5 | 1 | 16.67 | 0.852483 | -3.6633 | -5.258 | -2.1385 |
| APV | 9 | 1283 | 0.7 | -9.284449 | 1.8574 | 1.1948 | 2.5573 |
| AQA | 589 | 11 | 1.83 | -5.269893 | -6.8992 | -7.4758 | -6.2811 |
| AQE | 1 | 86 | 1.15 | -2.248905 | 1.2761 | -0.2379 | 2.8955 |
| AQG | 780 | 15 | 1.89 | -6.008875 | -6.9148 | -7.4542 | -6.3823 |
| AQP | 13 | 742 | 1.72 | -6.027823 | 0.9709 | 0.3642 | 1.5668 |
| AQQ | 1254 | 11 | 0.87 | -8.953354 | -7.6404 | -8.2637 | -7.073 |
| ARA | 704 | 483 | 40.69 | 43.4152 | -3.4585 | -3.7458 | -3.182 |
| ARD | 1 | 211 | 0.47 | -3.885231 | 1.991 | 0.5505 | 3.5284 |
| ARE | 2 | 332 | 0.6 | -4.788464 | 1.9041 | 0.7503 | 3.1407 |
| ARG | 5 | 465 | 1.06 | -5.297549 | 1.443 | 0.5853 | 2.3428 |
| ARL | 118 | 11 | 8.53 | 0.442914 | -5.3187 | -5.9608 | -4.7056 |
| ARP | 6 | 2142 | 0.28 | -12.705541 | 2.7163 | 1.9645 | 3.5222 |
| ARR | 6 | 1154 | 0.52 | -9.029309 | 3.9477 | 3.1408 | 4.7947 |
| ART | 1 | 1 | 50 | 2.281931 | -0.6365 | -2.8835 | 1.8105 |
| ARV | 462 | 1428 | 24.44 | 27.967699 | -0.0508 | -0.4784 | 0.3887 |
| ASR | 1 | 2 | 33.33 | 1.69879 | -0.1842 | -2.3735 | 1.9112 |
| ATR | 1 | 1 | 50 | 2.281931 | -0.6334 | -2.9578 | 1.696 |
| AVA | 2106 | 69 | 3.17 | -7.66256 | -4.5811 | -5.059 | -4.0949 |
| AVD | 28 | 1 | 3.45 | -0.828395 | -3.8056 | -5.2372 | -2.4966 |
| AVE | 647 | 11 | 1.67 | -5.676112 | -5.1419 | -5.829 | -4.4565 |
| AVH | 2012 | 28 | 1.37 | -10.507356 | -5.4081 | -5.9911 | -4.8753 |
| AVL | 376 | 1041 | 26.53 | 27.204126 | -0.16 | -0.5783 | 0.2939 |
| AVP | 5 | 579 | 0.86 | -6.095703 | 3.4418 | 2.59 | 4.4086 |
| AVR | 435 | 11 | 2.47 | -4.035965 | -4.7551 | -5.4673 | -4.056 |
| CLP | 227 | 2 | 0.87 | -3.807233 | -5.3734 | -6.4674 | -4.3258 |
| DAA | 885 | 11 | 1.23 | -7.128215 | -5.4466 | -6.1106 | -4.7289 |
| DEC | 158 | 3 | 1.86 | -2.71539 | -4.7991 | -5.8102 | -3.8025 |
| DHP | 1 | 344 | 0.29 | -5.084544 | 3.9906 | 2.7282 | 5.3245 |
| DLP | 2 | 120 | 1.64 | -2.457676 | 2.6843 | 1.5116 | 3.9654 |
| DPH | 1 | 459 | 0.22 | -5.930132 | 4.2378 | 2.971 | 5.5428 |
| DPP | 8 | 722 | 1.1 | -6.569294 | 3.2458 | 2.4942 | 4.0605 |
| DPR | 1 | 1 | 50 | 2.281931 | -0.6161 | -2.9191 | 1.6827 |
| DRG | 1 | 429 | 0.23 | -5.721558 | 4.1883 | 2.9608 | 5.5797 |
| DRH | 26 | 1 | 3.7 | -0.748929 | -3.7283 | -5.1904 | -2.4373 |
| DVL | 1 | 1 | 50 | 2.281931 | -0.622 | -2.8984 | 1.7519 |
| EAA | 1245 | 35 | 2.73 | -6.473264 | -4.7193 | -5.2606 | -4.193 |
| EAP | 2 | 228 | 0.87 | -3.817723 | 3.2512 | 2.1104 | 4.4348 |
| EEA | 1 | 436 | 0.23 | -5.770897 | 4.1876 | 2.8844 | 5.4773 |
| EGA | 5 | 907 | 0.55 | -7.970588 | 3.8612 | 2.9875 | 4.7274 |
| EGL | 1 | 135 | 0.74 | -2.995138 | 3.173 | 1.7999 | 4.5435 |
| EHA | 3 | 950 | 0.31 | -8.421396 | 4.2843 | 3.3123 | 5.3343 |
| EHG | 3 | 622 | 0.48 | -6.663089 | 3.8937 | 2.8987 | 4.9658 |
| ELP | 2 | 219 | 0.9 | -3.722296 | 3.2205 | 2.0743 | 4.446 |
| EPA | 315 | 214 | 40.45 | 28.775994 | -1.5642 | -2.016 | -1.109 |
| EPG | 3 | 435 | 0.68 | -5.415091 | 3.5571 | 2.5627 | 4.6359 |
| EPL | 1 | 2 | 33.33 | 1.69879 | -0.2041 | -2.3222 | 1.9747 |
| EPP | 4 | 666 | 0.6 | -6.783797 | 3.7392 | 2.8024 | 4.689 |
| EPQ | 1 | 2 | 33.33 | 1.69879 | -0.2055 | -2.3633 | 1.9428 |
| EPR | 2 | 984 | 0.2 | -8.699431 | 4.5652 | 3.4988 | 5.6918 |
| EQD | 3 | 360 | 0.83 | -4.827352 | 3.3972 | 2.3538 | 4.4594 |
| ERG | 3 | 741 | 0.4 | -7.3493 | 4.0459 | 3.0613 | 5.0463 |
| EVA | 635 | 12 | 1.85 | -5.451758 | -5.0503 | -5.7441 | -4.3783 |
| EVD | 2 | 174 | 1.14 | -3.205237 | 3.0088 | 1.8672 | 4.2502 |
| EVH | 997 | 11 | 1.09 | -7.72504 | -5.5653 | -6.2881 | -4.893 |
| EVL | 893 | 10 | 1.11 | -7.293207 | -5.5392 | -6.2355 | -4.8074 |
| EVP | 5 | 886 | 0.56 | -7.863643 | 3.8331 | 2.9261 | 4.6892 |
| EVV | 4 | 1 | 20 | 1.061191 | -1.972 | -3.8288 | -0.2853 |
| FRG | 1487 | 26 | 1.72 | -8.538134 | -5.1772 | -5.7321 | -4.6071 |
| FSI | 2 | 732 | 0.27 | -7.434228 | 4.3009 | 3.1885 | 5.4084 |
| FSL | 1 | 1 | 50 | 2.281931 | -0.6102 | -2.8803 | 1.7774 |
| GAA | 967 | 101 | 9.46 | 2.428059 | -3.4297 | -3.9001 | -2.9649 |
| GAG | 4 | 11 | 26.67 | 2.818323 | 2.4523 | 1.3629 | 3.5728 |
| GAL | 3 | 185 | 1.6 | -3.073566 | 5.4019 | 4.5238 | 6.3963 |
| GAP | 237 | 394 | 37.56 | 28.66777 | 2.1734 | 1.8479 | 2.5168 |
| GAR | 771 | 15 | 1.91 | -5.95177 | -2.2496 | -2.859 | -1.6881 |
| GEG | 6 | 620 | 0.96 | -6.213914 | 6.0675 | 5.3292 | 6.8125 |
| GET | 1 | 193 | 0.52 | -3.69349 | 5.9586 | 4.8703 | 7.1532 |
| GGA | 262 | 482 | 35.22 | 28.701242 | 2.275 | 1.9489 | 2.6058 |
| GGG | 155 | 113 | 42.16 | 21.545016 | 1.3426 | 0.9812 | 1.7394 |
| GGK | 112 | 464 | 19.44 | 10.883678 | 3.0806 | 2.7076 | 3.416 |
| GGM | 970 | 12 | 1.22 | -7.46923 | -2.6868 | -3.2991 | -2.0596 |
| GGP | 4 | 476 | 0.83 | -5.545336 | 6.1011 | 5.2818 | 6.9619 |
| GHD | 69 | 3 | 4.17 | -1.07385 | -1.4289 | -2.5854 | -0.3926 |
| GHH | 2 | 1 | 33.33 | 1.69879 | 0.4795 | -1.7045 | 2.6184 |
| GHR | 1 | 64 | 1.54 | -1.824793 | 4.9648 | 3.7654 | 6.204 |
| GLA | 153 | 2 | 1.29 | -2.935171 | -2.4651 | -3.6926 | -1.3549 |
| GLL | 882 | 15 | 1.67 | -6.626685 | -2.3811 | -2.9738 | -1.8275 |
| GLP | 6 | 176 | 3.3 | -2.152902 | 4.856 | 4.09 | 5.6305 |
| GLR | 203 | 5 | 2.4 | -2.79044 | -1.98 | -2.8703 | -1.1402 |
| GML | 2 | 354 | 0.56 | -4.970161 | 6.2231 | 5.2361 | 7.2452 |
| GPA | 282 | 583 | 32.6 | 28.028527 | 2.3925 | 2.0699 | 2.7158 |
| GPG | 3 | 3 | 50 | 3.95242 | 1.3138 | -0.2918 | 2.8295 |
| GPH | 4 | 198 | 1.98 | -2.978501 | 5.269 | 4.4104 | 6.139 |
| GPL | 1 | 1 | 50 | 2.281931 | 0.8401 | -1.6516 | 3.0775 |
| GPP | 1183 | 2376 | 33.24 | 58.299595 | 2.3653 | 2.0396 | 2.6473 |
| GPR | 6 | 1351 | 0.44 | -9.870991 | 6.8088 | 6.0619 | 7.5437 |
| GPS | 1 | 2 | 33.33 | 1.69879 | 1.5977 | -0.3889 | 3.6491 |
| GPV | 1 | 1 | 50 | 2.281931 | 0.8839 | -1.5284 | 3.2329 |
| GQG | 2 | 1 | 33.33 | 1.69879 | 0.4951 | -1.6792 | 2.7024 |
| GQH | 3 | 363 | 0.82 | -4.852179 | 6.0289 | 5.1776 | 6.9915 |
| GQP | 2 | 1 | 33.33 | 1.69879 | 0.4637 | -1.749 | 2.5752 |
| GRA | 2 | 136 | 1.45 | -2.698643 | 5.3474 | 4.3111 | 6.4087 |
| GRE | 3 | 58 | 4.92 | -0.765622 | 4.3014 | 3.4019 | 5.3477 |
| GRG | 4 | 2 | 33.33 | 2.402451 | 0.6666 | -1.0345 | 2.2624 |
| GRH | 2 | 192 | 1.03 | -3.420908 | 5.656 | 4.6492 | 6.7167 |
| GRP | 2 | 369 | 0.54 | -5.0904 | 6.2684 | 5.3133 | 7.2954 |
| GRQ | 3 | 811 | 0.37 | -7.724823 | 6.7768 | 5.9037 | 7.6623 |
| GVA | 956 | 12 | 1.24 | -7.394919 | -2.672 | -3.2861 | -2.0611 |
| GVG | 5775 | 89 | 1.52 | -17.392464 | -2.501 | -2.8578 | -2.1429 |
| GVL | 818 | 10 | 1.21 | -6.874182 | -2.6918 | -3.3525 | -2.0313 |
| GVP | 8 | 1202 | 0.66 | -9.03179 | 6.4774 | 5.7831 | 7.1255 |
| GVR | 3 | 1 | 25 | 1.328821 | 0.2226 | -1.9407 | 2.2046 |
| GVV | 9 | 1 | 10 | 0.30015 | -0.5891 | -2.4341 | 1.1215 |
| HAA | 2 | 1 | 33.33 | 1.69879 | 0.4822 | -1.685 | 2.5724 |
| HAG | 1512 | 17 | 1.11 | -9.483708 | -2.7911 | -3.3683 | -2.2734 |
| HAH | 1 | 1 | 50 | 2.281931 | 0.8692 | -1.5883 | 3.1286 |
| HAP | 2 | 307 | 0.65 | -4.573437 | 6.0944 | 5.1146 | 7.1029 |
| HAV | 4 | 366 | 1.08 | -4.687716 | 5.8542 | 5.0291 | 6.7246 |
| HGP | 17 | 122 | 12.23 | 2.117322 | 3.583 | 3.0405 | 4.1532 |
| HGQ | 1 | 688 | 0.15 | -7.32964 | 7.1017 | 6.0444 | 8.2081 |
| HHH | 265 | 3 | 1.12 | -3.965767 | -2.037 | -3.195 | -1.0852 |
| HHL | 2 | 2 | 50 | 3.227137 | 1.6581 | -0.2176 | 3.4478 |
| HHR | 6 | 573 | 1.04 | -5.905015 | 6.6533 | 5.9267 | 7.401 |
| HLA | 292 | 270 | 48.04 | 36.490463 | 2.2807 | 1.9507 | 2.5961 |
| HLH | 2833 | 30 | 1.05 | -13.107308 | -2.1749 | -2.6302 | -1.7104 |
| HLL | 191 | 1 | 0.52 | -3.671577 | -2.496 | -4.0059 | -1.164 |
| HLP | 101 | 1005 | 9.13 | 2.060621 | 4.6487 | 4.3114 | 5.0048 |
| HLR | 722 | 13 | 1.77 | -5.899222 | -1.6437 | -2.2584 | -1.0562 |
| HPA | 10 | 2295 | 0.43 | -12.880048 | 7.6031 | 6.9767 | 8.2194 |
| HPD | 544 | 7 | 1.27 | -5.551793 | -1.9577 | -2.7543 | -1.2287 |
| HPG | 2 | 11 | 15.38 | 1.079321 | 3.5704 | 2.3433 | 4.8346 |
| HPH | 95 | 576 | 14.16 | 6.547896 | 4.1528 | 3.8065 | 4.5189 |
| HPL | 7 | 388 | 1.77 | -4.322038 | 6.1598 | 5.4769 | 6.8829 |
| HPP | 5 | 461 | 1.07 | -5.267474 | 6.5751 | 5.7809 | 7.3578 |
| HPQ | 4 | 2 | 33.33 | 2.402451 | 1.1883 | -0.5925 | 2.7698 |
| HPR | 4 | 527 | 0.75 | -5.90252 | 6.8578 | 6.0625 | 7.6937 |
| HPV | 115 | 552 | 17.24 | 9.551726 | 3.9217 | 3.5662 | 4.2532 |
| HQG | 2 | 339 | 0.59 | -4.847 | 6.8314 | 5.9096 | 7.855 |
| HQH | 1 | 1 | 50 | 2.281931 | 1.2201 | -1.1923 | 3.6039 |
| HQL | 714 | 11 | 1.52 | -6.116021 | -1.7936 | -2.443 | -1.162 |
| HQP | 2 | 504 | 0.4 | -6.067673 | 7.1872 | 6.273 | 8.19 |
| HRA | 7 | 1076 | 0.65 | -8.563168 | 7.1409 | 6.4997 | 7.8906 |
| HRH | 1 | 3 | 25 | 1.328821 | 2.5157 | 0.672 | 4.316 |
| HRL | 1388 | 794 | 36.39 | 51.233366 | 1.8041 | 1.5246 | 2.1172 |
| HRP | 5 | 609 | 0.81 | -6.289664 | 6.8376 | 6.0725 | 7.616 |
| HRQ | 1 | 197 | 0.51 | -3.736936 | 6.5849 | 5.5187 | 7.7623 |
| HRR | 2 | 1 | 33.33 | 1.69879 | 0.8728 | -1.4617 | 3.0433 |
| HRV | 768 | 54 | 6.57 | -1.013034 | -0.2996 | -0.68 | 0.0902 |
| HTP | 4 | 1 | 20 | 1.061191 | 0.4525 | -1.7295 | 2.4308 |
| HTQ | 1258 | 18 | 1.41 | -8.258367 | -1.8753 | -2.4227 | -1.3466 |
| IGA | 903 | 19 | 2.06 | -6.270526 | -1.4933 | -2.0191 | -0.9639 |
| **ITA** | **1288** | **510** | **28.36** | **33.589892** | **1.4361** | **1.1361** | **1.7271** |
| ITP | 1730 | 33 | 1.87 | -8.972087 | -1.5907 | -2.0306 | -1.1501 |
| ITT | 1 | 1 | 50 | 2.281931 | 1.2292 | -1.347 | 3.5591 |
| KKV | 385 | 8 | 2.04 | -4.112779 | -1.5049 | -2.2692 | -0.7921 |
| KPA | 1 | 232 | 0.43 | -4.097755 | 6.7379 | 5.7274 | 7.9156 |
| LAA | 158 | 3 | 1.86 | -2.71539 | -1.5549 | -2.6373 | -0.515 |
| LAG | 33 | 1 | 2.94 | -1.009231 | -1.0934 | -2.7471 | 0.4496 |
| LAH | 4 | 1 | 20 | 1.061191 | 0.4428 | -1.7639 | 2.3821 |
| LAL | 2 | 68 | 2.86 | -1.474799 | 5.3333 | 4.3182 | 6.4202 |
| LAP | 2043 | 69 | 3.27 | -7.385656 | -1.0242 | -1.3852 | -0.6538 |
| LAR | 1 | 1 | 50 | 2.281931 | 1.1937 | -1.4259 | 3.4457 |
| LAS | 1 | 1 | 50 | 2.281931 | 1.2196 | -1.2966 | 3.58 |
| LAV | 3 | 2 | 40 | 2.759096 | 1.4044 | -0.4153 | 3.1143 |
| LDL | 1 | 1 | 50 | 2.281931 | 1.2216 | -1.3944 | 3.5039 |
| LDP | 1260 | 16 | 1.25 | -8.470938 | -1.9889 | -2.5433 | -1.4421 |
| LEP | 1771 | 23 | 1.28 | -9.999007 | -1.9699 | -2.4364 | -1.4619 |
| LGA | 293 | 5 | 1.68 | -3.815837 | -1.6852 | -2.5834 | -0.8637 |
| LGG | 2 | 1 | 33.33 | 1.69879 | 0.8727 | -1.5538 | 2.9238 |
| LGH | 393 | 4 | 1.01 | -4.911362 | -2.7933 | -3.8107 | -1.8142 |
| LGL | 866 | 10 | 1.14 | -7.14499 | -2.7329 | -3.5045 | -1.9526 |
| LGP | 318 | 6 | 1.85 | -3.859909 | -2.2436 | -3.1766 | -1.3382 |
| LGV | 811 | 8 | 0.98 | -7.087631 | -2.8804 | -3.7073 | -2.0224 |
| LHD | 1 | 1 | 50 | 2.281931 | 0.8853 | -1.6231 | 3.1473 |
| LHH | 3 | 8 | 27.27 | 2.489783 | 2.3731 | 1.0383 | 3.6395 |
| LHL | 4 | 2 | 33.33 | 2.402451 | 0.6706 | -1.0638 | 2.3426 |
| LHP | 213 | 6 | 2.74 | -2.674561 | -1.8588 | -2.816 | -0.9453 |
| LHQ | 57 | 2 | 3.39 | -1.198628 | -1.5807 | -2.9753 | -0.2918 |
| LHR | 707 | 657 | 48.17 | 57.022924 | 1.6002 | 1.0394 | 2.1204 |
| LKG | 15 | 188 | 7.39 | -0.059956 | 4.1344 | 3.4518 | 4.8999 |
| LLA | 3180 | 614 | 16.18 | 20.3067 | 0.0312 | -0.5367 | 0.5384 |
| LLD | 4 | 1 | 20 | 1.061191 | 0.00595 | -1.995 | 1.9547 |
| LLL | 21 | 1 | 4.55 | -0.526139 | -1.2629 | -2.9516 | 0.3518 |
| LLP | 1437 | 362 | 20.12 | 20.326015 | 0.2956 | -0.2576 | 0.8293 |
| LLQ | 3 | 166 | 1.78 | -2.82557 | 5.3062 | 4.3259 | 6.3945 |
| LLV | 638 | 7 | 1.09 | -6.185241 | -2.768 | -3.6338 | -1.8781 |
| LPA | 1111 | 98 | 8.11 | 0.799821 | -0.7561 | -1.3477 | -0.2047 |
| LPD | 1520 | 275 | 15.32 | 12.579288 | -0.0352 | -0.5909 | 0.4846 |
| LPG | 5 | 5 | 50 | 5.102552 | 1.4514 | 0.153 | 2.7634 |
| LPH | 220 | 5 | 2.22 | -3.005667 | -2.0511 | -3.057 | -1.1098 |
| LPL | 1174 | 260 | 18.13 | 15.284504 | 0.1664 | -0.3872 | 0.7086 |
| LPP | 32 | 2448 | 1.29 | -11.740684 | 5.9638 | 5.3477 | 6.5939 |
| LPQ | 1320 | 13 | 0.98 | -9.044361 | -2.9111 | -3.6597 | -2.183 |
| LPR | 685 | 28 | 3.93 | -3.622162 | -1.5215 | -2.1572 | -0.8788 |
| LPV | 2009 | 171 | 7.84 | 0.609862 | -0.7893 | -1.3579 | -0.2638 |
| LQA | 401 | 8 | 1.96 | -4.256809 | -2.2064 | -3.0445 | -1.3371 |
| LQH | 1 | 1 | 50 | 2.281931 | 0.859 | -1.5129 | 3.2702 |
| LQL | 5 | 333 | 1.48 | -4.202469 | 5.6107 | 4.745 | 6.5276 |
| LQP | 646 | 8 | 1.22 | -6.094285 | -2.6579 | -3.5123 | -1.8379 |
| LQR | 521 | 268 | 33.97 | 28.225555 | 1.0078 | 0.4577 | 1.5587 |
| LRA | 4199 | 70 | 1.64 | -14.537152 | -2.4122 | -3.0164 | -1.8556 |
| LRG | 844 | 237 | 21.92 | 18.005343 | 0.4041 | -0.1701 | 0.9283 |
| LRH | 292 | 3 | 1.02 | -4.227552 | -2.7457 | -3.8422 | -1.6451 |
| LRL | 2 | 1 | 33.33 | 1.69879 | 0.4785 | -1.8245 | 2.5779 |
| LRP | 3381 | 49 | 1.43 | -13.500083 | -2.5533 | -3.1684 | -1.9657 |
| LRR | 10 | 506 | 1.94 | -4.796843 | 5.4672 | 4.6969 | 6.2054 |
| LRT | 6 | 1 | 14.29 | 0.681621 | -0.2807 | -2.1628 | 1.598 |
| LRV | 1377 | 19 | 1.36 | -8.708365 | -2.5863 | -3.2658 | -1.9079 |
| LSL | 2 | 2 | 50 | 3.227137 | 1.186 | -0.7973 | 3.0414 |
| LSP | 1 | 8 | 11.11 | 0.411302 | 3.0528 | 1.5299 | 4.6148 |
| LTP | 11 | 1 | 8.33 | 0.109599 | -0.7348 | -2.5749 | 0.9701 |
| LVA | 422 | 4 | 0.94 | -5.14133 | -2.8523 | -3.8531 | -1.8283 |
| LVP | 6 | 26 | 18.75 | 2.416162 | 2.9918 | 2.0847 | 4.0058 |
| LVR | 306 | 2 | 0.65 | -4.56463 | -3.0785 | -4.3324 | -1.9233 |
| MKG | 1674 | 26 | 1.53 | -9.346308 | -2.4754 | -3.1319 | -1.806 |
| MLL | 1904 | 25 | 1.3 | -10.345127 | -2.644 | -3.3287 | -2.0097 |
| NSG | 2555 | 37 | 1.43 | -11.737774 | -2.5474 | -3.1494 | -1.9032 |
| PAA | 176 | 1267 | 12.2 | 6.773839 | 3.6438 | 3.0927 | 4.2002 |
| PAD | 2 | 2 | 50 | 3.227137 | -1.6921 | -3.5106 | 0.1853 |
| PAE | 7 | 25 | 21.88 | 3.087318 | -1.0257 | -1.8865 | -0.1988 |
| PAG | 42 | 853 | 4.69 | -3.188549 | 0.604 | 0.2519 | 0.9509 |
| PAH | 2 | 933 | 0.21 | -8.458614 | 3.4226 | 2.3727 | 4.5712 |
| PAL | 485 | 580 | 45.54 | 47.131661 | -2.2295 | -2.4486 | -2.0188 |
| PAP | 13 | 1720 | 0.75 | -10.668228 | 2.4453 | 1.8904 | 3.0048 |
| PAQ | 9 | 272 | 3.2 | -2.734844 | 1.0136 | 0.3498 | 1.7043 |
| PAR | 5 | 824 | 0.6 | -7.539237 | 2.6103 | 1.8204 | 3.4611 |
| PAV | 3375 | 717 | 17.52 | 24.339994 | -3.9585 | -4.1484 | -3.7652 |
| PDG | 2 | 16 | 11.11 | 0.581669 | -0.115 | -1.478 | 1.4038 |
| PDP | 3 | 295 | 1.01 | -4.255703 | 2.0716 | 1.1251 | 3.1706 |
| PEA | 2 | 650 | 0.31 | -6.973448 | 3.0973 | 2.0071 | 4.2574 |
| PEE | 2 | 1131 | 0.18 | -9.359029 | 3.5849 | 2.5585 | 4.7895 |
| PEP | 1 | 841 | 0.12 | -8.131742 | 3.6957 | 2.4924 | 5.0292 |
| PEQ | 5 | 640 | 0.78 | -6.484226 | 2.3698 | 1.5606 | 3.2003 |
| PER | 3 | 472 | 0.63 | -5.683321 | 2.5151 | 1.558 | 3.6002 |
| PEV | 2 | 1 | 33.33 | 1.69879 | -2.0697 | -4.0951 | 0.0638 |
| PGA | 6 | 1245 | 0.48 | -9.427315 | 2.8464 | 2.1087 | 3.6132 |
| PGG | 5 | 1159 | 0.43 | -9.158447 | 2.9301 | 2.1356 | 3.7495 |
| PGH | 3 | 2 | 40 | 2.759096 | -2.1769 | -3.8632 | -0.5929 |
| PGL | 1 | 288 | 0.35 | -4.617374 | 2.8086 | 1.4842 | 4.2141 |
| PGP | 7 | 1543 | 0.45 | -10.535474 | 2.911 | 2.2171 | 3.6354 |
| PGQ | 2 | 1168 | 0.17 | -9.517868 | 3.6189 | 2.5932 | 4.7525 |
| PGR | 4 | 1247 | 0.32 | -9.641999 | 3.1867 | 2.3403 | 4.1017 |
| PGV | 4 | 633 | 0.63 | -6.584987 | 2.5454 | 1.6982 | 3.4988 |
| PHA | 10 | 1858 | 0.54 | -11.428451 | 2.7706 | 2.1423 | 3.3984 |
| PHC | 1 | 3 | 25 | 1.328821 | -0.6484 | -2.7922 | 1.5819 |
| PHD | 5 | 280 | 1.75 | -3.682624 | 1.5936 | 0.7944 | 2.5172 |
| PHG | 6 | 612 | 0.97 | -6.16237 | 2.1687 | 1.3895 | 2.9529 |
| PHH | 8 | 965 | 0.82 | -7.908395 | 2.3446 | 1.6725 | 3.0584 |
| PHL | 8 | 884 | 0.9 | -7.487406 | 2.2546 | 1.61 | 2.9596 |
| PHP | 563 | 3063 | 15.53 | 18.350682 | -0.7154 | -0.9122 | -0.5207 |
| PHQ | 1 | 558 | 0.18 | -6.571754 | 3.3518 | 2.1132 | 4.7206 |
| PHR | 1082 | 1127 | 48.98 | 74.020187 | -2.3688 | -2.5773 | -2.1835 |
| PKH | 1 | 1 | 50 | 2.281931 | -1.2482 | -3.6053 | 1.208 |
| PKQ | 1 | 1 | 50 | 2.281931 | -1.2223 | -3.5991 | 1.2519 |
| PKR | 1 | 1 | 50 | 2.281931 | -1.244 | -3.6287 | 1.2762 |
| PLA | 2825 | 53 | 1.84 | -11.524998 | -6.3545 | -6.6825 | -6.0501 |
| PLD | 4 | 978 | 0.41 | -8.438473 | 2.9531 | 2.1105 | 3.9101 |
| PLG | 4 | 886 | 0.45 | -7.985778 | 2.8607 | 1.9767 | 3.7555 |
| PLH | 4 | 2 | 33.33 | 2.402451 | -2.5165 | -4.0102 | -0.9812 |
| PLL | 654 | 733 | 47.15 | 56.066471 | -2.2933 | -2.4948 | -2.0818 |
| PLP | 50 | 2130 | 2.29 | -9.229244 | 1.3443 | 1.0179 | 1.6728 |
| PLQ | 3 | 301 | 0.99 | -4.311485 | 2.112 | 1.0546 | 3.1347 |
| PLR | 6 | 581 | 1.02 | -5.958661 | 2.1226 | 1.3562 | 2.919 |
| PLV | 1346 | 24 | 1.75 | -8.07772 | -6.3678 | -6.8004 | -5.9446 |
| PPA | 586 | 5894 | 9.04 | 4.716404 | -0.1018 | -0.2988 | 0.0915 |
| PPD | 3 | 68 | 4.23 | -1.047592 | 0.7621 | -0.342 | 1.8755 |
| PPE | 3 | 3 | 50 | 3.95242 | -1.8995 | -3.4433 | -0.3057 |
| PPG | 140 | 1425 | 8.95 | 2.17135 | 0.7307 | 0.2371 | 1.2278 |
| PPH | 53 | 620 | 7.88 | 0.369532 | 0.8707 | 0.3493 | 1.4061 |
| PPL | 28 | 1709 | 1.61 | -9.316828 | 2.505 | 1.9117 | 3.0817 |
| PPP | 1307 | 6410 | 16.94 | 31.473123 | -0.00056 | -0.4982 | 0.4486 |
| PPQ | 1222 | 474 | 27.95 | 31.971622 | -2.5364 | -3.0114 | -2.0552 |
| PPR | 18 | 2896 | 0.62 | -14.105107 | 3.4481 | 2.7821 | 4.0468 |
| PPS | 1 | 4 | 20 | 1.061191 | 0.0594 | -1.9242 | 2.0374 |
| PPT | 1 | 10 | 9.09 | 0.200327 | 0.7478 | -0.9368 | 2.5705 |
| PPV | 27 | 3281 | 0.82 | -14.595013 | 3.1871 | 2.6031 | 3.7666 |
| PQA | 6 | 1241 | 0.48 | -9.410168 | 3.6204 | 2.8012 | 4.4672 |
| PQC | 1 | 1 | 50 | 2.281931 | -0.8138 | -3.1465 | 1.5486 |
| PQG | 3 | 370 | 0.8 | -4.909639 | 3.0435 | 2.0004 | 4.135 |
| PQH | 6 | 1010 | 0.59 | -8.361588 | 3.426 | 2.5817 | 4.2892 |
| PQL | 1036 | 53 | 4.87 | -3.299043 | -4.5432 | -5.0591 | -3.9973 |
| PQP | 17 | 1981 | 0.85 | -11.28398 | 3.1355 | 2.4741 | 3.7688 |
| PQQ | 2 | 1 | 33.33 | 1.69879 | -1.5438 | -3.5653 | 0.5635 |
| PQR | 1947 | 2004 | 49.28 | 99.702618 | -1.5612 | -2.0629 | -1.1175 |
| PQV | 305 | 9 | 2.87 | -3.11743 | -4.9761 | -5.752 | -4.2566 |
| PRA | 14 | 1195 | 1.16 | -8.372191 | 2.8216 | 2.135 | 3.5039 |
| PRD | 3 | 785 | 0.38 | -7.587492 | 3.7347 | 2.7526 | 4.823 |
| PRG | 4 | 1110 | 0.36 | -9.048906 | 3.8265 | 2.8275 | 4.7318 |
| PRH | 2 | 496 | 0.4 | -6.014136 | 3.5707 | 2.4369 | 4.7321 |
| PRL | 12 | 2655 | 0.45 | -13.823009 | 3.7467 | 3.0587 | 4.4327 |
| PRP | 65 | 3020 | 2.11 | -11.37258 | 2.2417 | 1.7273 | 2.7569 |
| PRQ | 3 | 1034 | 0.29 | -8.815877 | 3.987 | 2.9822 | 5.0478 |
| PRR | 6 | 791 | 0.75 | -7.231856 | 3.1884 | 2.3531 | 4.0301 |
| PRV | 734 | 16 | 2.13 | -5.579994 | -5.335 | -6.0049 | -4.7085 |
| PSP | 281 | 10 | 3.44 | -2.631802 | -4.8125 | -5.5592 | -4.0745 |
| PTP | 3 | 3 | 50 | 3.95242 | -1.2738 | -2.9124 | 0.2591 |
| PTV | 1 | 4 | 20 | 1.061191 | 0.0288 | -1.8582 | 2.1407 |
| PVA | 4 | 5 | 44.44 | 4.207934 | -1.1498 | -2.525 | 0.133 |
| PVE | 2 | 468 | 0.43 | -5.822925 | 3.5304 | 2.3675 | 4.708 |
| PVG | 4 | 1 | 20 | 1.061191 | -2.2833 | -4.0798 | -0.5931 |
| PVH | 7 | 40 | 14.89 | 1.92444 | 0.1965 | -0.6989 | 1.093 |
| PVL | 1098 | 459 | 29.48 | 32.928044 | -2.461 | -2.9393 | -1.9821 |
| PVP | 8 | 335 | 2.33 | -3.633605 | 2.1083 | 1.3243 | 2.9094 |
| PVQ | 2 | 877 | 0.23 | -8.186058 | 4.0955 | 3.008 | 5.2708 |
| PVR | 943 | 1939 | 32.72 | 51.403928 | -0.8691 | -1.3653 | -0.4167 |
| PVV | 1868 | 352 | 15.86 | 14.947409 | -3.2578 | -3.734 | -2.7834 |
| QAA | 459 | 9 | 1.92 | -4.580534 | -5.3811 | -6.1031 | -4.5821 |
| QAD | 2 | 1 | 33.33 | 1.69879 | -1.5422 | -3.6762 | 0.4293 |
| QAG | 2 | 142 | 1.39 | -2.784197 | 2.4803 | 1.2962 | 3.7691 |
| QAH | 1194 | 22 | 1.81 | -7.534212 | -5.5227 | -6.1333 | -4.9263 |
| QAL | 277 | 6 | 2.12 | -3.436075 | -5.2183 | -6.0234 | -4.3722 |
| QAP | 6 | 340 | 1.73 | -4.071959 | 2.3902 | 1.5299 | 3.2991 |
| QAV | 2 | 76 | 2.56 | -1.655053 | 1.9372 | 0.7295 | 3.3152 |
| QFA | 190 | 3 | 1.55 | -3.135975 | -5.3485 | -6.3838 | -4.4016 |
| QGQ | 1 | 2 | 33.33 | 1.69879 | -0.4465 | -2.5942 | 1.7368 |
| QHA | 956 | 10 | 1.04 | -7.628557 | -6.0024 | -6.7498 | -5.3156 |
| QHH | 1 | 1 | 50 | 2.281931 | -1.5547 | -3.951 | 0.9444 |
| QHL | 993 | 19 | 1.88 | -6.790793 | -6.935 | -7.4403 | -6.3971 |
| QHP | 860 | 504 | 36.95 | 41.294589 | -3.6094 | -3.9434 | -3.2858 |
| QHR | 2 | 174 | 1.14 | -3.205237 | 1.3652 | 0.1723 | 2.7269 |
| QHV | 786 | 7 | 0.88 | -7.074803 | -7.5318 | -8.2712 | -6.8466 |
| QLA | 4 | 1 | 20 | 1.061191 | -3.4009 | -5.1027 | -1.6963 |
| QLL | 2244 | 32 | 1.41 | -11.03796 | -7.2665 | -7.7256 | -6.8246 |
| QLP | 6 | 203 | 2.87 | -2.540834 | 0.4936 | -0.3589 | 1.3421 |
| QLV | 2104 | 30 | 1.41 | -10.688374 | -7.2635 | -7.7243 | -6.8072 |
| QPA | 8 | 638 | 1.24 | -6.042279 | 1.3015 | 0.6001 | 2.0729 |
| QPG | 1224 | 22 | 1.77 | -7.684962 | -7.0155 | -7.5259 | -6.5146 |
| QPH | 4 | 8 | 33.33 | 3.397579 | -2.0324 | -3.2491 | -0.7604 |
| QPL | 12 | 1 | 7.69 | 0.026325 | -4.5908 | -5.9908 | -3.2908 |
| QPP | 2 | 15 | 11.76 | 0.667593 | -0.713 | -2.1649 | 0.8294 |
| QPR | 6 | 955 | 0.62 | -8.092337 | 1.9563 | 1.1737 | 2.7937 |
| QQH | 3 | 315 | 0.94 | -4.43906 | 1.5393 | 0.4905 | 2.6175 |
| QQL | 4137 | 50 | 1.19 | -15.49145 | -7.4525 | -7.8694 | -7.0518 |
| QQR | 6 | 1 | 14.29 | 0.681621 | -3.8761 | -5.3796 | -2.3607 |
| QRA | 6 | 1037 | 0.58 | -8.490708 | 2.0371 | 1.2259 | 2.8617 |
| QVA | 236 | 4 | 1.67 | -3.431002 | -6.7674 | -7.6208 | -5.9303 |
| RAA | 830 | 20 | 2.35 | -5.697274 | -6.7145 | -7.2407 | -6.1934 |
| RAG | 1 | 73 | 1.35 | -2.00814 | 1.1591 | -0.3604 | 2.8551 |
| RAH | 2 | 98 | 2 | -2.088148 | 0.8561 | -0.3607 | 2.2915 |
| RAR | 1 | 5 | 16.67 | 0.852483 | -0.7349 | -2.7355 | 1.4778 |
| RAV | 2 | 1 | 33.33 | 1.69879 | -2.4823 | -4.4955 | -0.3209 |
| RDA | 2 | 324 | 0.61 | -4.720696 | 1.901 | 0.6871 | 3.1296 |
| RDR | 1 | 440 | 0.23 | -5.798903 | 2.5993 | 1.245 | 4.0558 |
| REH | 1 | 514 | 0.19 | -6.294647 | 2.7282 | 1.3136 | 4.1161 |
| REP | 3 | 418 | 0.71 | -5.287419 | 1.797 | 0.7324 | 2.8726 |
| RGA | 1 | 216 | 0.46 | -3.936859 | 2.012 | 0.5526 | 3.5352 |
| RGG | 2 | 658 | 0.3 | -7.019726 | 2.5126 | 1.3668 | 3.7154 |
| RGL | 5 | 1 | 16.67 | 0.852483 | -3.6525 | -5.238 | -2.0228 |
| RGM | 377 | 12 | 3.08 | -3.306134 | -6.3811 | -7.0057 | -5.7836 |
| RGP | 1 | 935 | 0.11 | -8.587498 | 3.2176 | 1.8734 | 4.5891 |
| RHA | 4 | 1231 | 0.32 | -9.574614 | 2.5556 | 1.6545 | 3.5446 |
| RHL | 1 | 3 | 25 | 1.328821 | -1.0382 | -3.2034 | 1.2017 |
| RHP | 1 | 1 | 50 | 2.281931 | -1.5406 | -3.9202 | 1.0911 |
| RHR | 1 | 275 | 0.36 | -4.502049 | 2.2052 | 0.7963 | 3.7342 |
| RKI | 1 | 257 | 0.39 | -4.337352 | 2.1546 | 0.7391 | 3.6914 |
| RLA | 1141 | 21 | 1.81 | -7.367587 | -6.983 | -7.4912 | -6.4571 |
| RLD | 1 | 1 | 50 | 2.281931 | -1.525 | -4.1067 | 0.9045 |
| RLG | 5 | 753 | 0.66 | -7.150109 | 1.8982 | 1.0326 | 2.8024 |
| RLH | 2 | 239 | 0.83 | -3.931343 | 1.6354 | 0.4572 | 2.962 |
| RLL | 3 | 2 | 40 | 2.759096 | -2.6848 | -4.305 | -0.8942 |
| RLP | 12 | 128 | 8.57 | 0.481311 | -0.6575 | -1.3206 | 0.0209 |
| RLQ | 1 | 544 | 0.18 | -6.484865 | 2.7734 | 1.4643 | 4.2101 |
| RLR | 4 | 1596 | 0.25 | -11.010233 | 2.7963 | 1.9094 | 3.7658 |
| RLV | 1229 | 13 | 1.05 | -8.63458 | -7.4744 | -8.0673 | -6.9043 |
| RPA | 3 | 270 | 1.1 | -4.015451 | 1.4082 | 0.3423 | 2.4782 |
| RPG | 2 | 573 | 0.35 | -6.511342 | 4.7278 | 3.6286 | 5.8803 |
| RPH | 463 | 52 | 10.1 | 2.237632 | -2.6376 | -3.1826 | -2.077 |
| RPL | 2 | 231 | 0.86 | -3.849029 | 3.9051 | 2.7899 | 5.0824 |
| RPP | 8 | 1331 | 0.6 | -9.589538 | 4.5256 | 3.7374 | 5.3216 |
| RPQ | 303 | 6 | 1.94 | -3.709505 | -4.2264 | -5.0735 | -3.3397 |
| RPR | 3 | 247 | 1.2 | -3.781891 | 3.6913 | 2.6466 | 4.7202 |
| RPS | 6 | 383 | 1.54 | -4.461115 | 3.5696 | 2.7078 | 4.4369 |
| RQG | 1 | 28 | 3.45 | -0.828395 | 2.4361 | 0.9685 | 3.9626 |
| RRH | 1 | 2 | 33.33 | 1.69879 | 0.2367 | -1.8783 | 2.2692 |
| RRL | 1 | 358 | 0.28 | -5.194814 | 4.6324 | 3.4362 | 5.9566 |
| RRV | 1 | 436 | 0.23 | -5.770897 | 4.8087 | 3.6087 | 6.1603 |
| RSA | 1 | 2 | 33.33 | 1.69879 | 0.2484 | -1.7517 | 2.4929 |
| RTA | 1 | 1 | 50 | 2.281931 | -0.2439 | -2.6311 | 1.966 |
| RTP | 1 | 1 | 50 | 2.281931 | -0.2571 | -2.5439 | 2.0323 |
| RVA | 9 | 21 | 30 | 4.678877 | 0.3938 | -0.4597 | 1.3301 |
| RVD | 987 | 21 | 2.08 | -6.529214 | -4.2643 | -4.9178 | -3.6521 |
| RVE | 2 | 1 | 33.33 | 1.69879 | -0.8247 | -2.876 | 1.2528 |
| RVH | 2 | 161 | 1.23 | -3.040661 | 3.5816 | 2.407 | 4.7194 |
| RVL | 635 | 16 | 2.46 | -4.884417 | -4.0826 | -4.7625 | -3.4241 |
| RVP | 2 | 302 | 0.66 | -4.529237 | 4.1442 | 3.004 | 5.2918 |
| RVV | 1093 | 26 | 2.32 | -6.5743 | -4.1618 | -4.7809 | -3.5545 |
| RWH | 1 | 881 | 0.11 | -8.328727 | 5.4187 | 4.2817 | 6.7196 |
| SAG | 3 | 190 | 1.55 | -3.135975 | 3.4588 | 2.4134 | 4.5199 |
| SAR | 1 | 1 | 50 | 2.281931 | -0.2206 | -2.5752 | 2.1106 |
| SKH | 534 | 2 | 0.37 | -6.2644 | -5.5063 | -6.6034 | -4.4148 |
| SPA | 2 | 1 | 33.33 | 1.69879 | -0.8205 | -3.0292 | 1.1563 |
| SPV | 8 | 1 | 11.11 | 0.411302 | -2.0949 | -3.8328 | -0.5592 |
| SSA | 9 | 1 | 10 | 0.30015 | -2.2125 | -3.8619 | -0.6245 |
| SSV | 1471 | 23 | 1.54 | -8.74696 | -4.573 | -5.2239 | -3.9631 |
| SWA | 1 | 67 | 1.47 | -1.887678 | 3.1912 | 1.8252 | 4.6123 |
| TLP | 1 | 1 | 50 | 2.281931 | -0.2431 | -2.5451 | 2.1301 |
| TPA | 4 | 2 | 33.33 | 2.402451 | -0.9682 | -2.5316 | 0.6625 |
| TPP | 1 | 4 | 20 | 1.061191 | 0.7925 | -0.9476 | 2.7635 |
| TRV | 2 | 1 | 33.33 | 1.69879 | -0.8206 | -2.9196 | 1.1841 |
| TSG | 963 | 12 | 1.23 | -7.43216 | -4.7514 | -5.4773 | -4.0259 |
| VAA | 4 | 19 | 17.39 | 1.801009 | 1.0644 | 0.0298 | 2.2487 |
| VAG | 8 | 2 | 20 | 1.500751 | -1.6484 | -3.1168 | -0.2771 |
| VAL | 1 | 1 | 50 | 2.281931 | -0.2305 | -2.5092 | 2.0844 |
| VAP | 3 | 1076 | 0.28 | -9.006677 | 5.0593 | 4.0959 | 6.0737 |
| VDR | 51 | 2 | 3.77 | -1.029977 | -3.3606 | -4.6151 | -2.174 |
| VFV | 1 | 1 | 50 | 2.281931 | -0.24 | -2.6042 | 2.0787 |
| VGG | 7 | 2 | 22.22 | 1.676846 | -1.5069 | -2.9923 | -0.0531 |
| VGP | 1 | 205 | 0.49 | -3.82237 | 4.147 | 2.8816 | 5.448 |
| VGR | 246 | 307 | 44.48 | 33.020401 | -0.2392 | -0.7513 | 0.2541 |
| VGV | 400 | 8 | 1.96 | -4.247925 | -4.2543 | -5.0365 | -3.4678 |
| VHA | 3 | 2 | 40 | 2.759096 | -0.7041 | -2.4033 | 0.9915 |
| VHP | 1 | 2 | 33.33 | 1.69879 | 0.2491 | -1.7743 | 2.389 |
| VHR | 615 | 353 | 36.47 | 34.216756 | -1.0159 | -1.5067 | -0.5266 |
| VKR | 2091 | 36 | 1.69 | -10.168798 | -4.4943 | -5.0591 | -3.92 |
| VLA | 2 | 2 | 50 | 3.227137 | -0.2798 | -2.0823 | 1.6216 |
| VLP | 5 | 192 | 2.54 | -2.644126 | 3.1208 | 2.1761 | 4.046 |
| VLQ | 1588 | 32 | 1.98 | -8.442363 | -4.2703 | -4.8963 | -3.6421 |
| VLV | 1232 | 14 | 1.12 | -8.545419 | -4.79 | -5.5069 | -4.0991 |
| VPA | 1831 | 245 | 11.8 | 7.441085 | -2.4062 | -2.9269 | -1.8393 |
| VPD | 2 | 1 | 33.33 | 1.69879 | -0.7788 | -2.8155 | 1.315 |
| VPG | 274 | 7 | 2.49 | -3.18782 | -3.9349 | -4.7914 | -3.0835 |
| VPH | 2 | 728 | 0.27 | -7.412412 | 4.9967 | 3.9091 | 6.1414 |
| VPL | 1 | 5 | 16.67 | 0.852483 | 1.0476 | -0.7226 | 2.9447 |
| VPP | 16 | 1961 | 0.81 | -11.294654 | 4.3515 | 3.6847 | 5.0867 |
| VPQ | 1 | 2 | 33.33 | 1.69879 | 0.2712 | -1.8252 | 2.3533 |
| VPR | 1 | 414 | 0.24 | -5.614376 | 4.8245 | 3.6334 | 6.1763 |
| VPV | 5 | 835 | 0.6 | -7.597782 | 4.5202 | 3.6014 | 5.4236 |
| VQA | 350 | 6 | 1.69 | -4.165277 | -4.3039 | -5.1859 | -3.4487 |
| VQP | 3489 | 63 | 1.77 | -12.957254 | -4.3922 | -4.9574 | -3.8265 |
| VQV | 1 | 1 | 50 | 2.281931 | -0.2105 | -2.5088 | 2.1108 |
| VRA | 8 | 2 | 20 | 1.500751 | -1.5971 | -3.0331 | -0.1451 |
| VRD | 1 | 237 | 0.42 | -4.14677 | 4.3295 | 3.0861 | 5.6891 |
| VRE | 1 | 2 | 33.33 | 1.69879 | 0.269 | -1.8118 | 2.4297 |
| VRG | 1671 | 61 | 3.52 | -6.285557 | -3.6932 | -4.2666 | -3.1122 |
| VRH | 1 | 154 | 0.65 | -3.240124 | 3.9586 | 2.6854 | 5.3664 |
| VRL | 290 | 104 | 26.4 | 14.240167 | -1.4189 | -2.0025 | -0.8754 |
| VRP | 9 | 797 | 1.12 | -6.880442 | 3.9943 | 3.2088 | 4.8169 |
| VRQ | 1 | 1 | 50 | 2.281931 | -0.2212 | -2.4988 | 2.1513 |
| VRR | 1078 | 26 | 2.36 | -6.490263 | -4.0875 | -4.7218 | -3.4516 |
| VRV | 4 | 3 | 42.86 | 3.551605 | -0.5884 | -2.1118 | 0.8584 |
| VVL | 1 | 3 | 25 | 1.328821 | 0.601 | -1.3484 | 2.5651 |
| VVR | 67 | 50 | 42.74 | 14.469924 | -0.6834 | -1.3382 | -0.0671 |
| VWR | 1 | 1 | 50 | 2.281931 | -0.2073 | -2.4545 | 2.162 |
| WAE | 1 | 17 | 5.56 | -0.313206 | 2.077 | 0.5397 | 3.6587 |
| WEE | 3 | 867 | 0.34 | -8.012688 | 4.9199 | 3.9347 | 5.9755 |
| WRG | 1 | 452 | 0.22 | -5.882124 | 4.9059 | 3.6838 | 6.2153 |
| YGC | 1155 | 18 | 1.53 | -7.756975 | -4.502 | -5.152 | -3.8124 |
| YGS | 5 | 1 | 16.67 | 0.852483 | - | - | - |

Frequency of occurrence of every sequence observed in the screening of the mutagenesis performed on the TM region of *hokC* presenting both wild type (WT) and mutant (Mutant) phenotypes. From these frequencies, the observed incorrect sequence-phenotype assignments rates (100*Mutant Frequency/[WT Frequency + Mutant Frequency]) is shown in the table in the column labeled “**Observed ISPAs (%)**”. The expected rate values according to two statistical methods (BiM and ByM, see Methods) are indicated in the columns labeled “**BiM Z-score (α=5%)**” and “**ByM Mean”**, “**ByM HPD lower limit**” or “**ByM HPD upper limit**”; in the ByM columns; HPD stands for Highest Posterior Density. The data for the wild-type sequence are highlighted in bold. For the BiM any sequence with Z-score <= 1.65 or in the case of the ByM any positive mean value has a frequency of ISPAs that may be explained by the expected rate of error from experimental/technical reasons with 95% of confidence.

**Supplementary References**

15. Poulsen, L.K., Refn, A., Molin, S. and Andersson, P. (1991) Topographic analysis of the toxic Gef protein from Escherichia coli. *Molecular microbiology*, **5**, 1627-1637.

31. Punta, M., Coggill, P.C., Eberhardt, R.Y., Mistry, J., Tate, J., Boursnell, C., Pang, N., Forslund, K., Ceric, G., Clements, J. et al. (2012) The Pfam protein families database. *Nucleic acids research*, **40**, D290-301.
